# Deuterated Molecular Probes for Metal Sensing

**DOI:** 10.1021/acsomega.6c03570

**Published:** 2026-08-01

**Authors:** Grysette Daher, Maria Daniela Santi, Leander K. May, Lisa M. Fries, Stefan Glöggler

**Affiliations:** † NMR Signal Enhancement Group, Max Planck Institute for Multidisciplinary Sciences, Am Fassberg 11, 37077 Göttingen, Germany; ‡ Center for Biostructural Imaging of Neurodegeneration, University Medical Center, Von-Siebold-Str. 3A, 37075 Göttingen, Germany; § Advanced Imagining Research Center, University of Texas Southwestern Medical Center, 5723 Forest Park Rd, Dallas, Texas 75390, United States; ∥ Department of Biomedical Engineering, University of Texas Southwestern Medical Center, 5323 Harry Hines Blvd, Dallas, Texas 75390, United States

## Abstract

Deuterium is generating a lot of interest due to its relevance in, e.g., drug discovery. It is also arising as a new tool to design molecular probes for nuclear magnetic resonance (NMR), offering advantages as it can be quantified and referenced with respect to natural abundant deuterium in water. Herein, we introduce the concept of using deuterium as a readout for metal sensing. Metals play an important role in biology and new sensing capabilities pave the pathways to a better understanding of their role in health and diseases. In particular, we developed a Zn^2+^ sensor based on selectively deuterated tris (2-pyridylmethyl)­amine (TPA) for which we introduce isotopic labeling strategies. The probe selectively detected Zn^2+^ over other physiologically relevant ions, which include copper, iron, calcium, potassium, and sodium, and was sensitive down to 100 μM. *T*_1_ measurements for bound (36.6 ms for pyridine and 41.0 ms for aliphatic deuterons) and unbound TPA-*d*_9_ showed a 10-fold difference with water (366.6 ms), making it suitable for fast averaging sequences in potential MRI applications.

## Graphical abstract

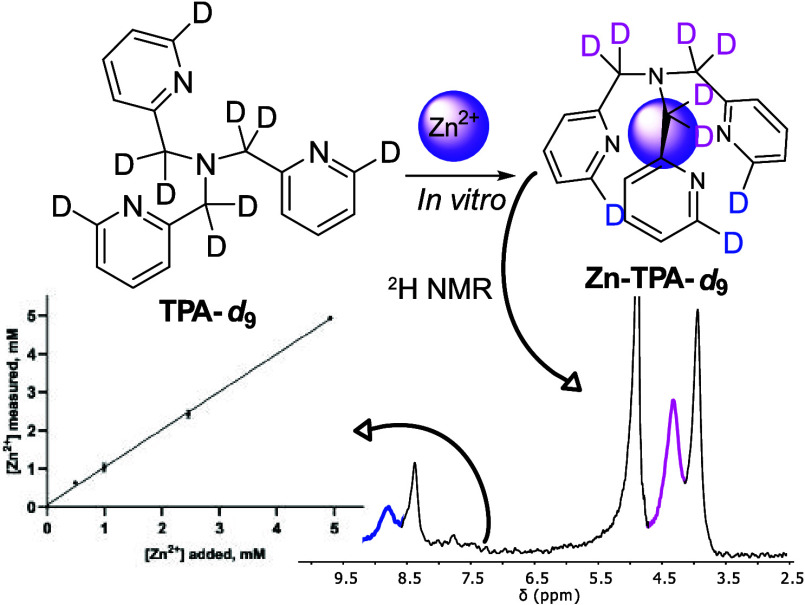

## Introduction

Using deuterium as a molecular probe to study biological systems is an emerging methodology that has created increasing interest because of the introduction of deuterium metabolic spectroscopy (DMS) and imaging (DMI).^1^ The use of deuterated molecular probes is a simple and robust approach to mapping metabolism. Early reports in this field have been focused on deuterated glucose to study bacterial metabolism,^2,3^ synthesis of liver glycogen using [6,6-^2^H_2_]-glucose,^4^ or metabolism in red blood cells.^5^ *In vivo* deuterium spectroscopy studies using [6,6-^2^H_2_]-glucose were recently reported in the rat brain at 16.4 T, achieving high-resolution ^2^H nuclear magnetic resonance (NMR).^6,7^ In 2018, the DMI concept was introduced utilizing [6,6-^2^H_2_]-glucose ^2^H NMR to image glucose, glutamate + glutamine (Glx), and lactate in rat and human brains.^8^ Apart from [6,6-^2^H_2_]-glucose, other deuterated molecular probes were used for *in vivo* studies,^9^ including perdeuterated glucose,^10,11^ fumarate,^12^ acetate,^13^ and fatty acids.^9^ Furthermore, 2-deoxy-2-[^2^H_2_]-d-glucose^14^ was proven to function similarly to 2-[^18^F]-fluoroglucose in positron emission tomography (PET) to study glucose metabolism dysregulation in diseases such as cancer and neurodegenerative disorders in the brain. In addition to studying metabolism, new possibilities have opened up to develop molecular probes for imaging experiments. For example, deuterated nanopolymers based on G5-polyamidoamine (PAMAM) dendrimers labeled with deuterated acetylated groups were used for *in vivo* maps of lymph node activity in inflammatory processes,^15^ or deuterated thymine (thy-*d*_3_) as a reporter probe/reporter gene system via its accumulation as monophosphate in kinase 1­(hTK1) transgene.^16^

Another relevant field of research is the detection of metal ions. Here, we introduce the concept of designing deuterated molecular probes for metal sensing. In this proof-of-concept study, we focus on synthesizing and assessing a deuterated Zn^2+^ probe. Zinc ions are relevant because they are involved in many cellular processes, such as enzyme catalysis, intracellular signaling, and neurotransmission.^17–20^ Zn^2+^ sensors investigated in the past mainly made use of paramagnetic species, with even dual functional MRI and fluorescent sensors being reported.^21–28^ In addition, spectroscopic approaches exploring fluorine^29^ NMR and hyperpolarization^30,31^ have been pursued. From this, hyperpolarized ^15^N-deuterated tris­(2-pyridylmethyl)­amine (TPA) was reported to quantify freely available Zn^2+^ ions in human prostate tissues and intact cells via chemical shift of the central amino nucleus.^31^ Herein, we report the synthesis and in-phantom characterization of a selectively deuterated TPA molecule as a Zn^2+^ sensor. Compared with the existing techniques, deuterium probes offer the advantage of being referenced against the intrinsic deuterated water signal of tissue (about 16.6 mM)^15^ and thereby providing a quantitative readout, and, in comparison to hyperpolarized ^13^C or ^15^N nuclei, possibilities for an extended imaging window.

## Results and Discussion

Based on tetradentate amino-containing groups applicable for Zn^2+^ sensing, as described in a ^15^N hyperpolarization study,^31^ we hypothesized that changes in chemical shift can be perceived in the protons adjacent to the binding pocket. Therefore, the substitution of these for deuterons could be used for quantification of Zn^2+^. When the fully protonated TPA molecule coordinates Zn^2+^, all proton signals present a downfield shift in the ^1^H NMR (Figure 1). Protons in the region around 7–8 ppm are particularly attractive since they are spectrally well separated from the HDO signal (4.79 ppm). We found that the ortho pyridine proton at 8.25 ppm displays a 0.53 ppm difference between the bound and unbound Zn^2+^ complex and was thus chosen as a site for H/D exchange accompanied by the aliphatic signal at 3.62 ppm, with a chemical shift difference of 0.58 ppm. Larger chemical shift differences on the order of 1 ppm are typically desirable. However, it has been shown that peaks can be successfully separated *in vivo* ^32^ with chemical shift ranges similar to those observed for TPA, which encouraged us to pursue the investigation of deuteration strategies.

**Figure 1:**
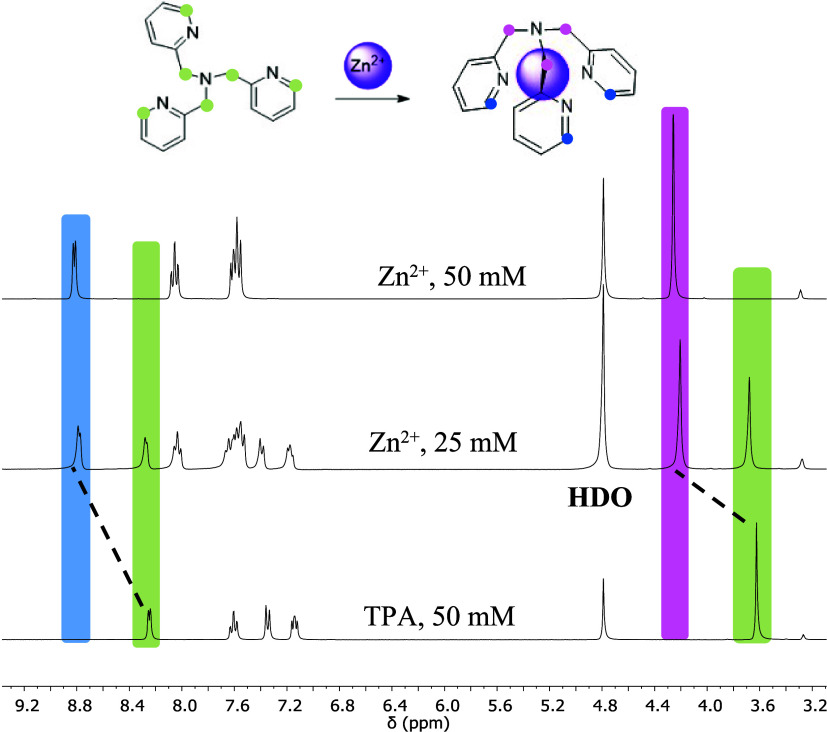
^1^H NMR of TPA (300 MHz, D_2_O) with various amounts of zinc chloride (ZnCl_2_). Unbound TPA protons are marked in green, aliphatic protons of the Zn-TPA complex are marked in pink, and 2-C pyridine protons on the Zn-TPA complex are marked in blue.

Initial screening under acidic and basic conditions (Table 1, entries 1 and 2) was conducted to evaluate site-selective deuteration of TPA. In both cases, the α-position to the central amino group showed the most reactivity with high deuteration percentages for DCl and low deuteration for NaOD, while the pyridine scaffold stayed unreacted. N-oxidized pyridine is a frequently used scaffold for activation of the C-2 position;^33,34^ thus, we explored deuteration reactions in per-N-oxidized TPA (see SI, Scheme S1B). Deuteration attempts on this molecule with traditional basic and acidic conditions (see SI, Table S1, items 1 and 2) demonstrate an inherent instability, producing a mixture of inseparable products. Lowering the temperature of the reaction (from 100 to 50 °C, see SI, Table S1, items 4 and 5) did not improve the complex, crude mixture. Sodium carbonate salt (see SI, Table S1, item 3) showed no deuteration, recovering the starting material, while potassium carbonate and *t*-butoxide showed complex decomposition products (see SI, Table S1, items 6 and 7). A similar strategy, using N-oxidized TPA-*d*_6_ (see SI, Scheme S1C), was tested. Full N-oxidation of TPA-*d*_6_ was not achieved, but rather two inseparable mixtures of products were obtained, **S2.1** and **S2.2** (see SI, Scheme S1C), products of incomplete N-oxidation in the central amino group. This mixture of products was used for deuteration under basic conditions (NaOD, D_2_O, 100 °C), and the crude mixture showed a complex profile. The observed instability is consistent with previous reports describing the reduced stability of N-oxidized pyridines, attributed to the formation of the N^+^–O^–^ bond, which can perturb aromaticity and weaken adjacent bonds.^35^

**Table 1:**
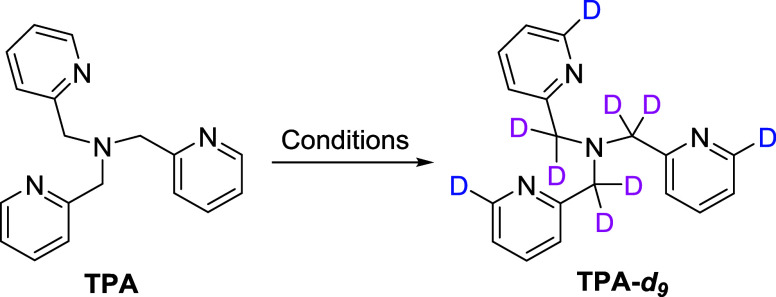
Screening of Selective Deuteration Conditions on the TPA Molecule

item	conditions	*o*-pyridine D (%)	α-position D (%)	yield (%)
1	NaOD/D_2_O/100 °C^a^	0	12	–
2	DCl/D_2_O/100 °C	0	99	96
3	RuCl_2_[PPh_3_]_3_/KOD/D_2_O/Zn/80 °C	0	0	–
4	RuCl_2_[PPh_3_]_3_/NaOD/D_2_O/Zn/80 °C	0	0	–
5	KO*^t^* Bu/DMSO-*d*_6_/100 °C	0	95	–
6	K_2_CO_3_/DMSO-*d*_6_-D_2_O/18-crown-6/80 °C	0	16	–
7	Ru_3_(CO)_12_/*^t^* BuOD/115 °C	0	65	–
8	NaBD_4_/Pd/C/D_2_O/150 °C	73	95	26
9	NaBD_4_/Pd/C/D_2_O/100 °C	20	50	–
10	NaBD_4_/Pd/C–Pt/C/D_2_O/100 °C	0	0	–

a – Not isolated. Note: Deuterium incorporation was calculated by comparing the integral of the residual proton signal against a reference proton signal from the TPA molecule. For entry 8, a fully protonated solution of TPA was used as a reference signal in the same solvent and concentration as the deuterated product. Yield was determined as the ratio of the isolated product amount against the expected amount, in mol.

Driven by the previous results, we transitioned to the use of metal catalysis, specifically ruthenium catalysis, which has been shown effective for H/D exchange of molecules with amino groups.^36,37^ Catalytic amounts of RuCl_2_[PPh_3_]_3_ have been proven to promote selective deuteration with a variety of functional groups through the delicate alteration of additives and cocatalysts^37^ using D_2_O as the deuterium source. Similarly, Ru_3_(CO)_12_ has also been reported as a catalyst for selective deuteration^36^ using *t*-BuOD as a source of deuterium. Items 3, 4, and 7 from Table 1 display the results of these conditions applied to the TPA molecule. The selective deuteration on the pyridine ring was not observed for items 3 and 4 of Table 1, and the starting material was recovered. While Ru_3_(CO)_12_ (Table 1, item 7) proved mildly efficient for the deuteration of the α-position of the central amino group with a 65% deuterium incorporation. We attribute the lack of reactivity to the chelating ability of TPA to interact with Ru, thus deactivating its catalytic effect. Other basic conditions were tested^38,39^ (Table 1, items 5 and 6), where DMSO-*d*_6_ serves as a deuterium source via *in situ* dimsyl anion^39^ formation, which was shown to be able to deprotonate the *ortho* position of the pyridine ring. In both cases, the α-position to the central amino group was deuterated, with the absence of deuterium in the pyridine ring. We furthermore explored the direct use of deuteride and discovered that, when TPA is reacted with catalytic amounts of NaBD_4_ and Pd/C in D_2_O,^40^ the selective deuteration of TPA was enabled (Table 1, items 8–10). A small screening of different conditions was tested to improve the yield. Lowering the temperature to 100 °C (Table 1, item 9) provided a low deuterium incorporation, while using a mixture of Pd/C–Pt/C (Table 1, item 10) showed no deuterium incorporation in any position, and the starting material was recovered. In addition to obtaining the desired product using the reaction with NaBD_4_ and Pd/C in D_2_O, a prominent secondary byproduct was observed, identified as **S-3** (see SI, Section 8), in which cleavage of the central N–C bond is observed. Therefore, TPA-*d*_9_ was synthesized with a 26% yield and deuteration of 73% for the pyridine 2-C position and 95% of the amino α-position.

To evaluate ion selectivity, TPA-*d*_9_ was challenged against a variety of physiologically relevant metal ions and citrate under identical conditions (5 mM in 100 mM HEPES buffer, pH 7.0). Cu^2+^ was excluded due to its paramagnetic properties. No chemical shift changes were observed in any of the competition experiments, as shown in Figure 2 (see SI, Figure S3 for ^2^H NMR).

**Figure 2:**
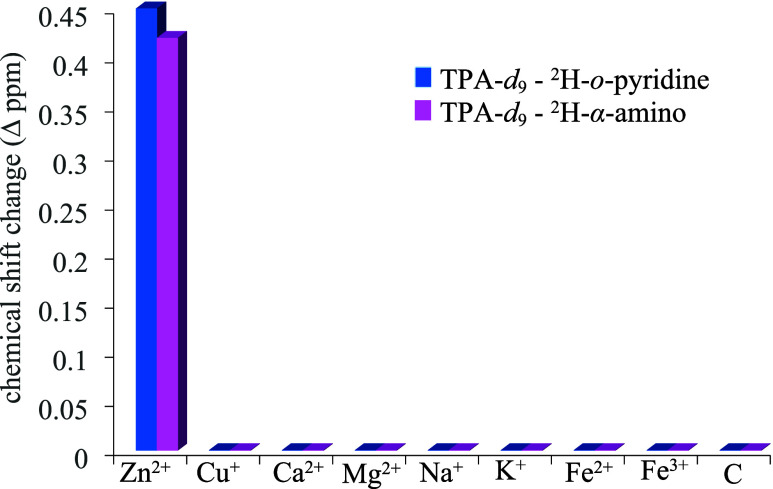
Specificity of TPA-*d*_9_ (5 mM) in 100 mM HEPES buffer 7.0 with physiologically relevant cations and citrate (C) on the chart (5 mM of each).

For assessing TPA-*d*_9_ as a probe for zinc ions, aqueous solutions in HEPES buffer pH 7.0 (5 mM TPA-*d*_9_) were prepared. Increasing amounts of ZnCl_2_ were added and ^2^H NMR spectra were obtained at 14.1 T. In agreement with initial tests on fully protonated TPA, TPA-*d*_9_ displayed well-resolved resonances with a linear response to different amounts of Zn^2+^, as shown in Figure 3. Pyridine deuterons display a smaller signal than the aliphatic deuteron, primarily because there is only one nucleus that contributes (in comparison to 2 from both geminal deuterons in the sp^3^ bond) to the signal and second because the deuterium incorporation is lower (73%) compared with the aliphatic position (95%). The quantification of zinc ions can be achieved by determining the ratio of the bound and unbound signals from either the aliphatic or the pyridine deuteron signals. Since the total concentration of the probe is known, eq S1 can be applied (see SI, Section 5.2 for more details). For the 5 mM TPA-*d*_9_ samples with 0.5 mM Zn^2+^, the lowest zinc concentration studied, a total of 800 scans was sufficient to observe the bound and unbound signals on the aliphatic deuteron. In contrast, the pyridine deuteron signal is significantly lower; therefore, increasing the number of scans to 1650 (not optimized for scan time based on *T*_1_, see below: around 45 min, in comparison to 20 min, see SI, Figure S6) can make up for that intensity loss and a clearer signal was obtained. Longer acquisitions of up to 4800 scans (∼2 h) were additionally performed and showed the same result after quantification. In this region, an accurate quantification of Zn^2+^ can be obtained, as shown by the linear response in Figure 3. In addition, the limit of quantification (LOQ) of each signal was below 0.5 mM (0.489 mM for aliphatic deuteron and 0.464 mM for pyridine, see SI, Section 5.3), and the limit of detection (LOD) for both signals was below 0.2 mM (0.161 mM for aliphatic deuteron and 0.153 mM for pyridine, see SI, Section 5.3).

**Figure 3:**
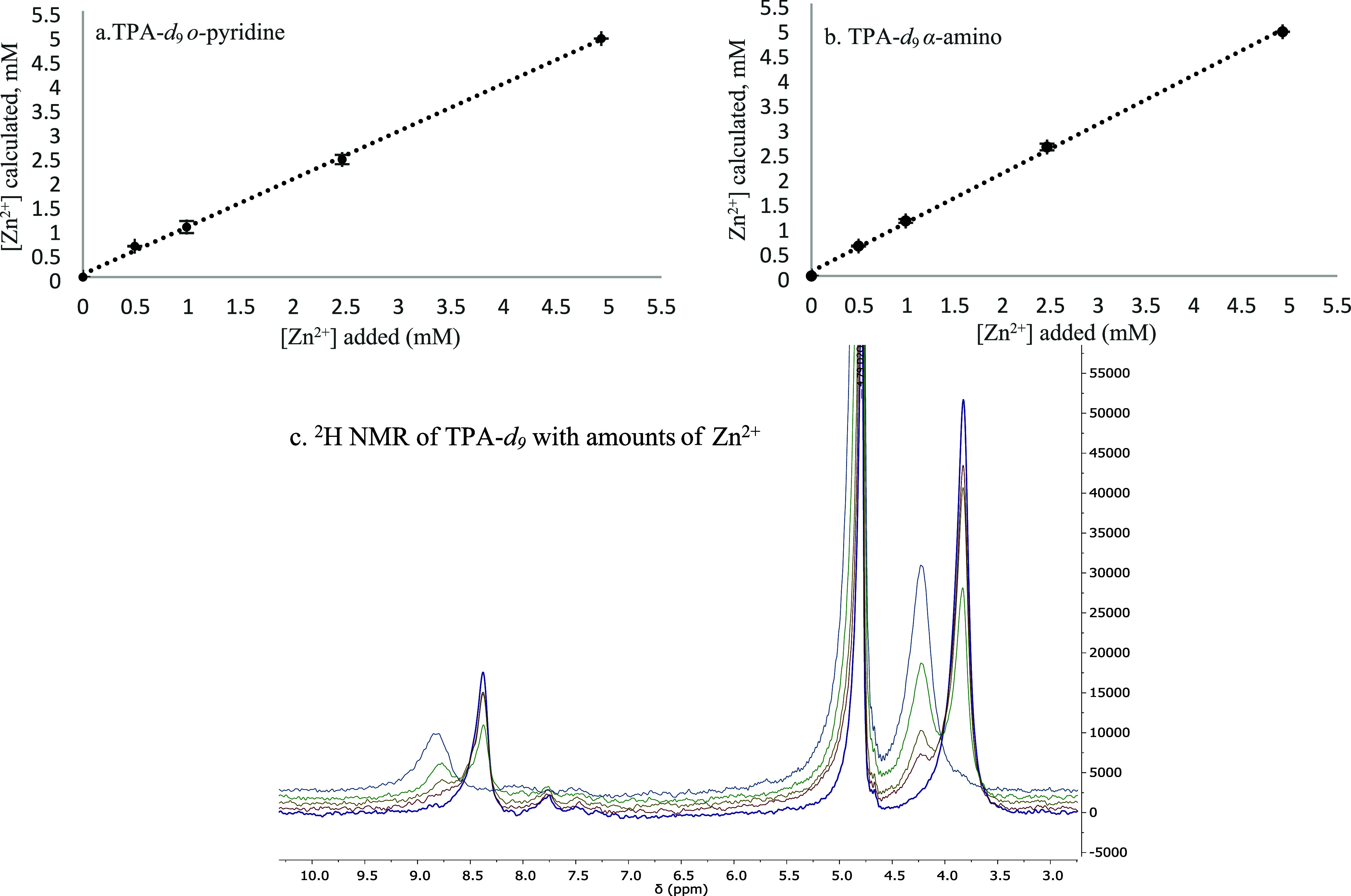
(a) Plot of [Zn^2+^] as determined by ^2^H NMR of *o*-pyridine deuteron versus the known amount of Zn^2+^ added to each sample. (b) A plot of [Zn^2+^] as determined by ^2^H NMR of α-amino deuteron versus the known amount of Zn^2+^ added to each sample. (c) ^2^H NMR of TPA-*d*_9_ (600 MHz) in 100 mM HEPES buffer pH 7.0 with decreasing amounts of zinc chloride (ZnCl_2_) from top to bottom: 5.0, 2.5, 1.0, and 0.5 mM. [Zn^2+^] quantification was determined by using eq S1 from SI, which calculates the zinc concentration based on the zinc-complexed TPA-*d*_9_ integral as a fraction of the sum of the bound and unbound integrals.

For potential biological applications, lower probe concentrations are beneficial to avoid toxicity or perturbation of processes in which Zn^2+^ is involved. Although the probe enables dual-modality Zn^2+^ sensing through both pyridine and aliphatic deuteron resonances, the substantially higher sensitivity of the intense aliphatic signal makes it more suitable for measurements at lower probe concentrations. Using this resonance, the concentration of the deuterated probe could be reduced 5-fold (to 1 mM TPA-*d*_9_), while still allowing Zn^2+^ detection down to 0.1 mM at 600 MHz with a signal-to-noise ratio (SNR) above 10, suitable for reliable quantification, as shown in Figure 4. Given the possibility of working with a low probe concentration, we decided to apply our methodology to cells (see SI, Section 9). While it is possible to observe the probe signal in the cell suspension (applying the probe in a DMSO solution), the possibility of distinguishing between bound and unbound signals is hindered by line broadening of the deuterium signal. Further discussion of the application in cells can be found in the Supporting Information.

**Figure 4:**
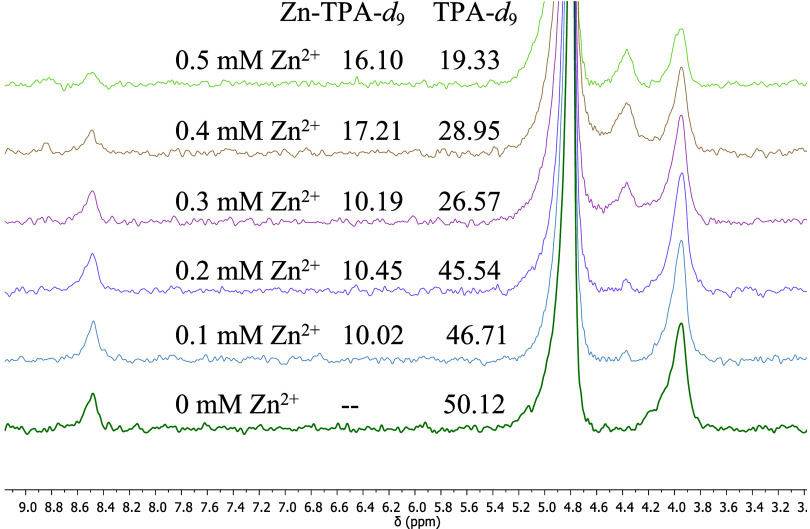
^2^H NMR of TPA-*d*_9_ (1 mM, 600 MHz) in 100 mM HEPES buffer pH 7.0 with decreasing amounts of zinc chloride (ZnCl_2_) and SNR values for bound and unbound signals of aliphatic deuterons. SNR above 3 is suitable for detection, while SNR equal to or higher than 10 is suitable for quantification. SNR was calculated using TopSpin.

The sensitivity demonstrated by this proof-of-concept probe is comparable to those reported previously. The highest sensitivities are shown by hyperpolarized probes,^30,31^ with detection limits as low as 5 ^31^ and 200 μM in phantom images,^30^ while porphyrin fluorescence sensors^28^ can estimate in the nM range. Probes with intermediate sensitivity consisting of gadolinium or manganese complexes display a range of sensitivity between 60 and 500 μM.^21–28^ This TPA-*d*_9_ probe achieves a highly competitive LOD of 100 μM with a low probe concentration of 1 mM (lower than in most of the reports) for a deuterium-based NMR technique.^8,9,14^ Therefore, this probe is not designed to measure low-level intracellular free Zn^2+^, even when the TPA molecule is membrane permeable. Rather, it is more suitable for noninvasive detection of relatively high and dynamic labile zinc pools present during physiological states (e.g., benign prostatic hyperplasia)^41^ or pathological conditions associated with elevated zinc levels (e.g., prostate cancer, Alzheimer’s disease, ischemic stroke, and inflammation).^41–44^

The longitudinal relaxation times (*T*_1_) for TPA-*d*_9_, Zn-TPA-*d*_9_ complex, and HDO for each sample were measured in 100 mM HEPES buffer at pH 7.0, and the results are summarized in Table 2. The short *T*_1_ times, on the order of 30–40 ms, of deuterium generally allow for a rapid acquisition to increase the SNR.^45^

**Table 2:** Relaxation Times of TPA-*d*_9_, Zn-TPA-*d*_9_ Complex, and HDO of Each Sample at 14.1 T (600 MHz, ^2^H)

compound	^2^H-*o*-pyridine *T*_1_ (ms)	^2^H-α-amino *T*_1_ (ms)	water *T*_1_ (ms)
TPA-*d*_9_	34.7	33.1	426.9
Zn-TPA-*d*_9_	36.6	41.0	366.6

## Conclusions

In conclusion, we introduce deuterated metal sensors and demonstrate their Zn^2+^ ion selectivity *in vitro*. To accomplish this, optimization of deuteration strategies was necessary to obtain the TPA-*d*_9_ probe. The synthetic pathway to selectively deuterate TPA in the 2-C position of the pyridine group as well as the α-position of the central amino group was described. The newly deuterated probe exhibited selectivity only in the presence of zinc, and not when exposed to competing ions or citrate. When Zn^2+^ ions are present at a physiologically relevant pH, TPA-*d*_9_ (at 1 mM) proves effective in detecting concentration changes down to 100 μM, well within the range of physiological Zn^2+^ concentrations in tissues such as the prostate (∼2.5 mM), pancreas β-cells (∼10 to 20 mM), and brain (∼1 mM).^46–48^ The proof-of-concept studies presented here highlight the potential of deuterated probes for metal sensing. Future developments will focus on chemical modifications in the structure of TPA or its derivatives, inducing a larger chemical shift (>1 ppm). Since the aliphatic position showed the most promising, targeting a diagnostic signal away from the residual water peak without losing selectivity for zinc ions is attractive, thereby facilitating detection in future *in vivo* studies. Overall, we see great potential of deuterated molecular probes for a variety of first applications such as prostate cancer detection.^31^ Besides Zn^2+^ sensors, the concept can be expanded to any metal, including the metal ions discussed here, with molecules that have a different selectivity.

## Supplementary Material



## Notes

Details of the synthetic procedures and synthesis of TPA-*d*_9_, NMR of all synthesized products, ^2^H NMR of the TPA-*d*_9_ probe with a zinc ion, relaxation time measurements, selectivity test, and additional cell data (PDF)
