# Supplementary material for: Deuterated Molecular Probes for Metal Sensing
Source: ACS Omega. 2026 Aug 1;11(31):46340–6. doi: 10.1021/acsomega.6c03570 (PMC13470902; doi:10.1021/acsomega.6c03570)
Supplement: Supplementary file 1 [file ao6c03570_si_001.pdf]

ELECTRONIC SUPPORTING INFORMATION

Deuterated molecular probes for metal sensing

Grysette Daher[a,b], Maria Daniela Santi,[a,b] Leander K. May,[a,b] Lisa M. Fries[a,b], Stefan Glöggler*[a,b,c,d]

[a]*NMR Signal Enhancement Group, Max Planck Institute for Multidisciplinary Sciences Göttingen, Germany;* [b]*Center for Biostructural Imaging of Neurodegeneration, University Medical Center Göttingen, Germany;* [c]*Advanced Imagining Research Center, University of Texas Southwestern Medical Center 5723 Forest Park Rd, TX75390, United States.* [d] *Department of Biomedical Engineering, University of Texas Southwestern Medical Center, 5323 Harry Hines Blvd*, *Dallas, Texas 75390, United States*

Table of Contents

1. **General considerations** **S3**
2. **Synthetic procedure** **S4**
   - 2.1 General procedure for deuteration with NaOD **S4**
   - 2.2 General procedure for deuteration with DCl **S4**
   - 2.3 General procedure for deuteration with Na_2CO_3 **S4**
   - 2.4 General procedure for deuteration with K_2CO_3 **S4**
   - 2.5 General procedure for deuteration with KO^tBu **S5**
   - 2.6 Deuterations described in table 1 from the main text **S5**
   - Tris(pyridin-2-ylmethyl-*d*$_2$) amine. **TPA-*d*$_6$** **S6**
   - Tris((1-oxopyridin-2-yl)methyl)amine oxide. **S1** **S6**
   - Tris((pyridin-2-yl-6-*d*)methyl-*d*$_2$)amine. **TPA-*d*$_9$** **S7**
3. **Approach for selective deuteration of TPA** **S10**
4. **Deuterations on *N*-oxidized-TPA** **S10**
5. **Quantification of Zn^{2+}** **S11**
6. **T_1 relaxation time of TPA-*d*$_9$ and Zn-TPA-*d*$_9$** **S13**
7. **Selectivity test on TPA-*d*$_9$** **S14**
8. **Products identified with $NaBD_4$ deuteration** **S15**
9. **Cell experiments** **S17**
10. **^1H, ^2H and ^{13}C NMRs of new compounds** **S20**

1. General considerations

Chemicals and reagents were purchased from commercial suppliers and used as received. ^{1}H, ^{2}H and ^{13}C were recorded on an Avance IV (NEO) wide bore system (Bruker Biospin GmbH, Karlsruhe) operated at 300.13 MHz ^{1}H frequency, equipped with a narrow bore shim insert (BOSS/3). The hard 90° pulses on this system were 10.16 µs for ^{1}H, 10.0 µs for ^{13}C and 275 µs for ^{2}H. Probe temperature was 298 K. TopSpin 4.0.7 was used for acquisition on this system.

Zinc experiments (^{2}H) were measured on an AVANCD NEO, probe CPP TCI 600S3 H&F-C/N-D-05 Z ET with ATMA at 14.1 T (^{2}H: 92.1 MHz). Probe temperature was 298 K. TopSpin 4.0.7 was used for acquisition on this system. The hard 90° pulse on this system was 75 µs for ^{2}H. Each point describes on section 5.1 item III were measured with the following parameters: NS: 800; RG: 1.78; D1: 1 s; acquisition time: 0.5 s.

Cell experiments (^{2}H) were measured on an AVANCD NEO, probe CPP TCI 1H/19F-13C/15ND Z-Grad, Z135422/0002 at 16.4 T (^{2}H: 107.5 MHz). Probe temperature was 310 K. TopSpin 4.0.4 was used for acquisition on this system. The 90° Hard pulse were used with this probe with 100 µs for ^{2}H. Each point was measured with the following parameters: NS: from 400 to 1200 ; RG: 101; D1: 1 s; acquisition time: 0.5 s

The signals of the deuterated solvents were used as internal chemical shift standards. Residual proton signals from the deuterated solvents were used as references as follows: for ^{1}H: D_2O (4.79 ppm), methanol-d_4 (3.31 ppm); for ^{2}H: D2O (4.79 ppm), methanol-d_4 (3.31 ppm); for ^{13}C: methanol-d_4 (49.05 ppm). All coupling constants were measured in Hertz.

LC-MS analysis was performed with HPLC ThermoScientific – Ultimate 3000 consisting of a UV detector (DAD-3000, pump (HPG-3200SD), autosampler (WPS3000), column oven (TCC-3000) and ISQEM mass spectrometer. HPLC was performed with the following conditions: column: Phenomenex Kinetex C18 1.7 µm 50 x 2.1mm, flow rate: 0.5 mL/min, mobile phase: acetonitrile 0.1% formic acid and water 0.1% formic acid in gradient from 2:98 to 50:50 in 4 min. Mass spectra (MS) were recorded on ThermoScientific LTQ XL linear ion trap with ESI positive mode. High resolution mass spectra were obtained on Exactive Plus, 2.9 Build 290492 by Thermo Fisher Scientific. Analytical TLCs were performed on aluminium-backed TLC plates and visualized with

UV light (254 nm). Flash column chromatography was performed using silica gel (230-400 mesh, 60 Å) purchased from Sigma Aldrich. All moisture-sensitive reactions were carried out in oven-dried glassware using nitrogen industrial-grade cylinders.

2. Synthetic procedure

2.1 General procedure for deuteration with NaOD

In a glass pressure tube, suitable starting material, either TPA or *N*-oxidized TPA **S1**, (1 equiv.) was dissolved in D_2O [1 M] at room temperature followed by the addition of NaOD (40% in D_2O) (2 equiv.). The tube was sealed and the crude mixture was stirred at 100 °C for 12 h. After the reaction was completed, the pH was adjusted to 7-8 with HCl 1 M aq. solution (10 drops). The solvent was evaporated under reduced pressure, and the compound was isolated by silica gel chromatography using the corresponding mobile phase.

2.2 General procedure for deuteration with DCl

In a glass pressure tube, suitable starting material, either TPA or *N*-oxidized TPA **S1**, (1 equiv.) was dissolved in D_2O [1 M] at room temperature followed by the addition of DCl (38% in D_2O) (1 equiv.). The tube was sealed and the crude mixture was stirred at 100 °C for 12 h. After the reaction was completed, the pH was adjusted to 7-8 with NaOH 1 M aq. solution (10 drops). The solvent was evaporated under reduced pressure, and the compound was isolated by silica gel chromatography using the corresponding mobile phase.

2.3 General procedure for deuteration with Na_2CO_3

In a glass pressure tube, suitable starting material, either TPA or *N*-oxidized TPA **S1**, (1 equiv.) was dissolved in D_2O [1 M] at room temperature followed by the addition of Na_2CO_3 (1 equiv.). The tube was sealed and the crude mixture was stirred at 100 °C for 12 h. After the reaction was completed, the pH was adjusted to 7-8 with HCl 1 M aq. solution (10 drops). The solvent was evaporated under reduced pressure, and the compound was isolated by silica gel chromatography using the corresponding mobile phase.

2.4 General procedure for deuteration with K_2CO_3

In a glass pressure tube, suitable starting material, either TPA or *N*-oxidized TPA **S1**, (1 equiv.) was dissolved in D_2O [1 M] at room temperature followed by the addition of K_2CO_3 (1 equiv.). The tube was sealed and the crude mixture was stirred at 100 °C for 12 h. After the reaction was completed, the pH was adjusted to 7-8 with HCl 1 M aq. solution (10 drops). The solvent was evaporated under reduced pressure, and the compound was isolated by silica gel chromatography using the corresponding mobile phase.

2.5 General procedure for deuteration with KO^tBu

In a glass pressure tube, suitable starting material, either TPA or *N*-oxidized TPA **S1**, (1 equiv.) was dissolved in DMSO-d_6 [0.5 M] at room temperature followed by the addition of KO^tBu (1 equiv.). The crude mixture was stirred at room temperature for 12 h. After the reaction was completed, an aliquot of the crude was subject to NMR analysis.

2.6 Deuterations described in table 1 from the main text were performed as follow:

2.6.1 Deuteration using **$RuCl_2[PPh_3]_3$**, was performed as described by Plietker[1], briefly: $RuCl_2[PPh_3]_3$ (2.5 mol%) and Zinc (2 equiv.) were added under nitrogen stream into an oven-dried rounded bottom flask, followed by the addition of TPA dissolved in 1,4-dioxane and KOD in D_2O (25 mol%, 40 wt% in D_2O). The crude mixture was stirred at 80 °C for 12 h. The procedure was performed similarly for NaOD (25 mol%, 40 wt% in D_2O).

2.6.2 Deuteration using **KO^tBu**, was performed as described by Gao[2], briefly: In a pressure-resistant Schlenk tube were added KO^tBu (1 equiv.) and TPA (1 equiv.) under a nitrogen stream, then DMSO-d_6 (17 equiv.) was added, the Schlenk tube sealed and heated to 100 °C for 12 h.

[1] Eisele, P.; Ullwer, F.; Scholz, S.; Plietker, B. Mild, Selective Ru-Catalyzed Deuteration Using D2O as a Deuterium Source. *Chem. - A Eur. J.* **2019**, *25* (72), 16550–16554. https://doi.org/10.1002/chem.201904927.

[2] Li, Y.; Zheng, C.; Jiang, Z. J.; Tang, J.; Tang, B.; Gao, Z. Potassium Tert-Butoxide Promoted Regioselective Deuteration of Pyridines. *Chem. Commun.* **2022**, *58* (21), 3497–3500. https://doi.org/10.1039/d1cc07128a

2.6.3 Deuteration using **$Ru_3(CO)_{12}$**, was performed as described by Mihovilovic[3], briefly: In a pressure tube TPA (1 equiv.), *t*-BuOD (5 equiv. per deuteration position) and $Ru_3(CO)_{12}$ (2.5 mol%) were added and the tube was flushed with nitrogen several times, then the tube was sealed and heated to 140 °C for 12 h.

2.6.4 Deuteration using **$NaBD_4$**, was performed as described by Brückner[4], in which a pressure tube is filled with nitrogen, next TPA (1 equiv.), Pd/C (10 wt%, 10 mol%), $NaBD_4$ (10 mol%) and D_2O [0.16 M] were added, the tube sealed and heated in an oil bath to 150 °C for 72 h.

Tris(pyridin-2-ylmethyl-d$_2$) amine. TPA-*d*$_6$

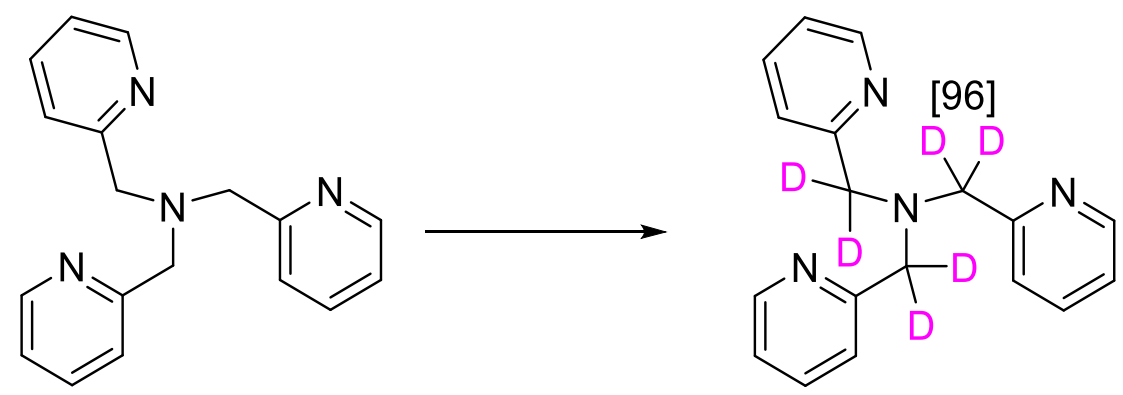

TPA-*d*$_6$ was synthesized following the general procedure: 2.2. The crude mixture was purified by column chromatography using a mobile phase of CH_3CN(9)/H_2O(1), yielding compound **TPA-*d*$_6$** (99%, 96% D, 419 mg) as a white solid: ^{1}H NMR (300 MHz, Methanol-*d*$_4$) δ 8.46 (d, *J* = 5.0 Hz, 1H), 7.79 (dt, *J* = 7.6, 3.5 Hz, 1H), 7.61 (d, *J* = 7.6 Hz, 1H), 7.33 – 7.13 (m, 1H), 3.86 (s, 0.5H). ^{2}H NMR (46 MHz, Methanol-*d*$_4$) δ 3.86 (s, 1H). ^{13}C NMR (76 MHz, Methanol-*d*$_4$) δ 159.40, 149.54, 138.82, 124.96, 124.00, 60.06 (q, t, *J* = 22.65 Hz). LCMS (ESI+) calculated for $C_{18}H_{12}D_6N_4$ $[M+H]^+$ 297.19 experimental value 297.27.

[3] Gröll, B., Schnürch, M., Mihovilovic, M. D. *Selective Ru(0)-Catalyzed Deuteration of Electron-Rich and Electron-Poor Nitrogen-Containing Heterocycles, J. Org. Chem.* **2012**, 77, 4432−4437. dx.doi.org/10.1021/jo300219v |

[4] Derdau, V.; Atzrodt, J.; Zimmermann, J.; Kroll, C.; Brückner, F. Hydrogen-Deuterium Exchange Reactions of Aromatic Compounds and Heterocycles by $NaBD_4$-Activated Rhodium, Platinum and Palladium Catalysts. *Chem. - A Eur. J.* **2009**, *15* (40), 10397–10404. https://doi.org/10.1002/chem.200901107.

Tris((1-oxopyridin-2-yl)methyl)amine oxide. S1

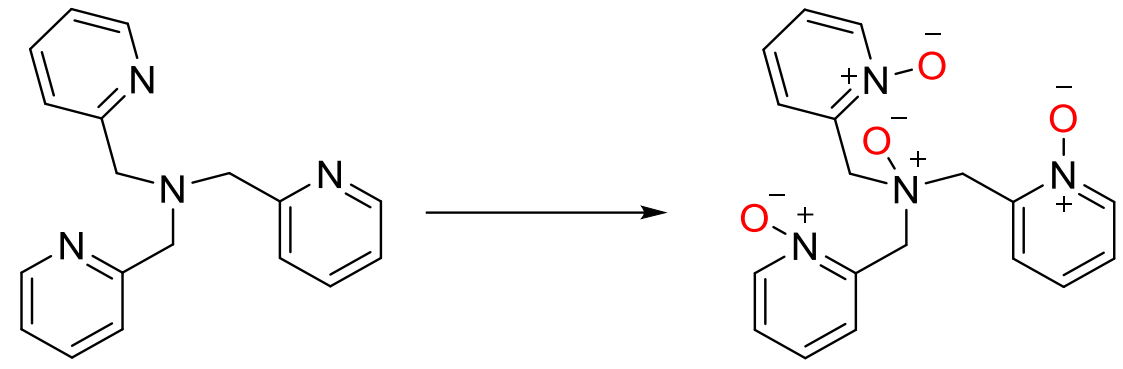

TPA (1 equiv., 396 mg) was dissolved in dry DCM (6 mL) and placed in an ice bath with magnetic stirring under nitrogen atmosphere. Then *m*-chloroperoxybenzoic acid (4 equiv., 1.4 g) was added. When the bubbling stopped, the ice bath was removed, and the crude mixture was stirred at room temperature for 12 h. After the reaction was completed, methanol was added (5 mL), then silica gel and the solvent carefully removed under reduced pressure. The crude mixture was purified by column chromatography using a gradient of Hex(9)/AcOEt(1) to elute the benzoic acid and then MeOH(9)/H_2O(1) yielding compound **S-1** (79%, 382 mg) as a white solid: ^{1}H NMR (300 MHz, Methanol-d_4) δ 8.38 (dd, J = 6.0, 1.8 Hz, 1H), 7.87 (dd, J = 7.3, 2.8 Hz, 1H), 7.54 (qt, J = 7.7, 3.3 Hz, 2H), 5.01 (s, 2H). ^{13}C NMR (76 MHz, Methanol-d_4) δ 142.74, 140.99, 133.92, 129.05, 128.88, 65.41. LCMS (ESI+) calculated for $C_{18}H_{18}N_4O_4$ $[M+H]^+$ 355.13 experimental value 355.20.

Tris((pyridin-2-yl-6-*d*)methyl-*d*$_2$)amine. TPA-*d*$_9$

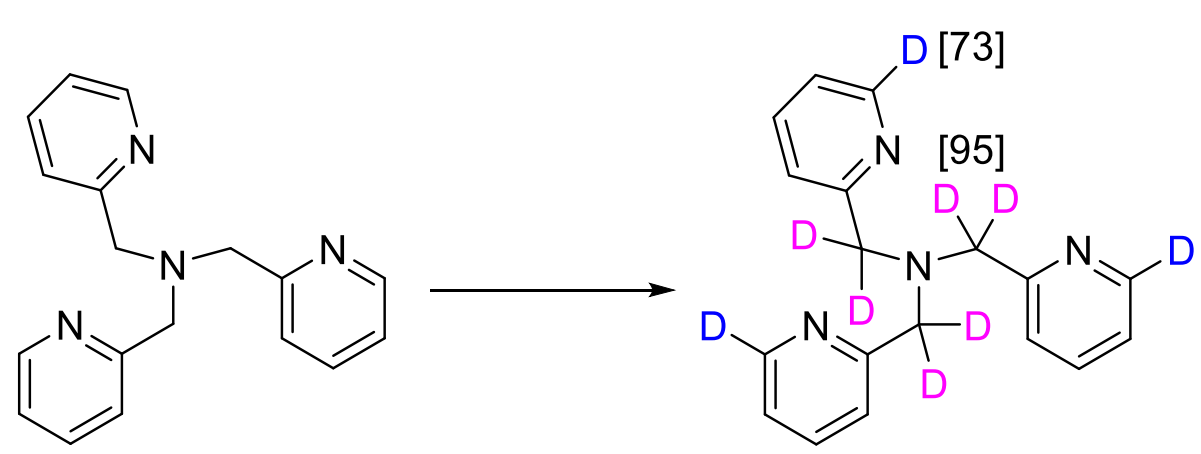

TPA-d_9 was synthesized following procedure 2.6.4. The crude mixture was purified by column chromatography using a gradient from CH_2Cl_2(8)/EtOAc(2) to CH_2Cl_2(9)/CH_3OH(1) yielding compound **TPA-*d*$_9$** (26%, 409 mg) as a white solid: ^{1}H NMR (300 MHz, Methanol-d_4) δ 8.43 (d, J = 5.0 Hz, 1H), 8.00 – 7.71 (m, 1H), 7.66 (d, J = 7.1 Hz, 1H), 7.40 – 7.13 (m, 1H), 3.82 (s, 2H). ^{2}H NMR (46 MHz, Methanol-d_4) δ 8.46 (s, 1H), 3.82 (s, 2H). ^{13}C NMR (76 MHz, Methanol-d_4) δ

159.96, 152.16 – 146.25 (t, J = 28.15 Hz), 138.65, 124.89, 123.72, 61.79 – 57.60 (q, J = 19.9 Hz). HRMS (ESI+) calculated for $C_{18}H_{10}D_9N_4$ $[M+H]^+$ 300.2174 experimental value 300.2169.

Tris((1-oxidopyridin2-yl)methyl-*d*$_2$)amine oxide-1,1-*d*$_2$ (S2.1) and 2,2´,2´´-(nitrilotris(methylene-*d*$_2$))tris(pyridine 1-oxide) (S2.2)

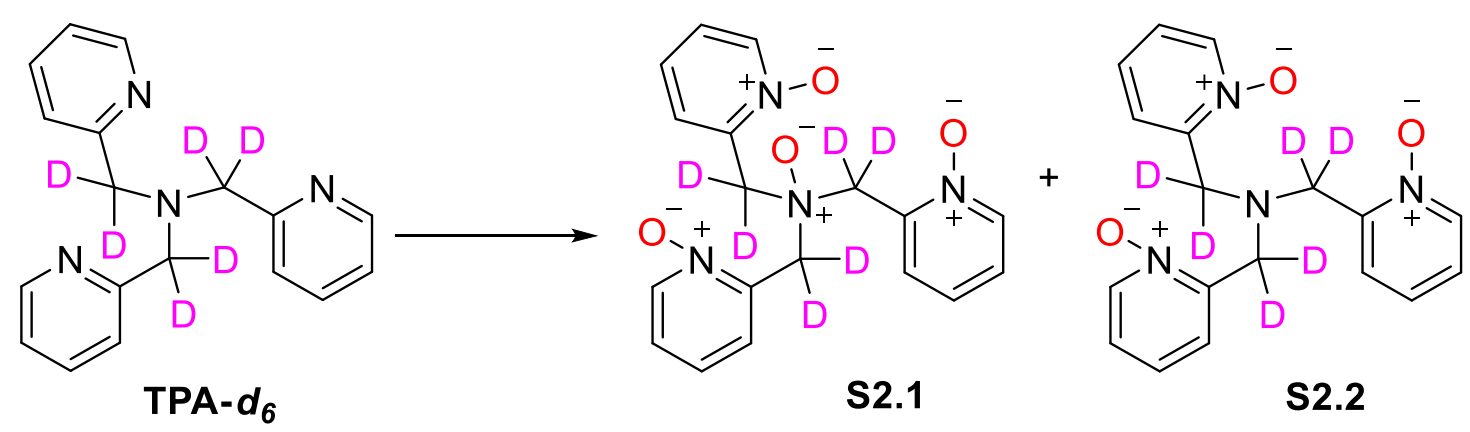

TPA-d_6 (1 equiv., 396 mg) was dissolved in dry DCM (6 mL) and placed in an ice bath with magnetic stirring under nitrogen atmosphere. Then *m*-chloroperoxybenzoic acid (4 equiv., 1.3 g) was added. When the bubbling stopped, the ice bath was removed, and the crude mixture was stirred at room temperature for 12 h. After the reaction was completed, methanol was added (5 mL), then silica gel and the solvent carefully removed under reduced pressure. The crude mixture was purified by column chromatography using a gradient of Hex(9)/AcOEt(1) to elute the benzoic acid and then MeOH(9)/H_2O(1) yielding compound **S2.1 and S2.2** (79%, 363 mg, inseparable mixture) as a white solid: ^1H NMR (300 MHz, D_2O) δ 8.65 (s, 1H), 8.55 – 8.37 (m, 2H), 7.82 (s, 1H), 7.69 (dd, J = 6.3, 3.3 Hz, 2H), 7.63 – 7.49 (m, 1H), 5.05 (s, 1H). ^{13}C NMR (76 MHz, D_2O) δ 142.15, 141.97, 141.49, 133.87, 131.90, 129.86, 129.74, 64.39. LCMS (ESI+) calculated for $C_{18}H_{18}N_4O_4$ (Compound **S2.1**) $[M+H]^+$ 361.17 experimental value 361.31. LCMS (ESI+) calculated for $C_{18}H_{12}D_6N_4O_3$ (Compound **S2.2**) $[M+H]^+$ 345.18 experimental value 345.25.

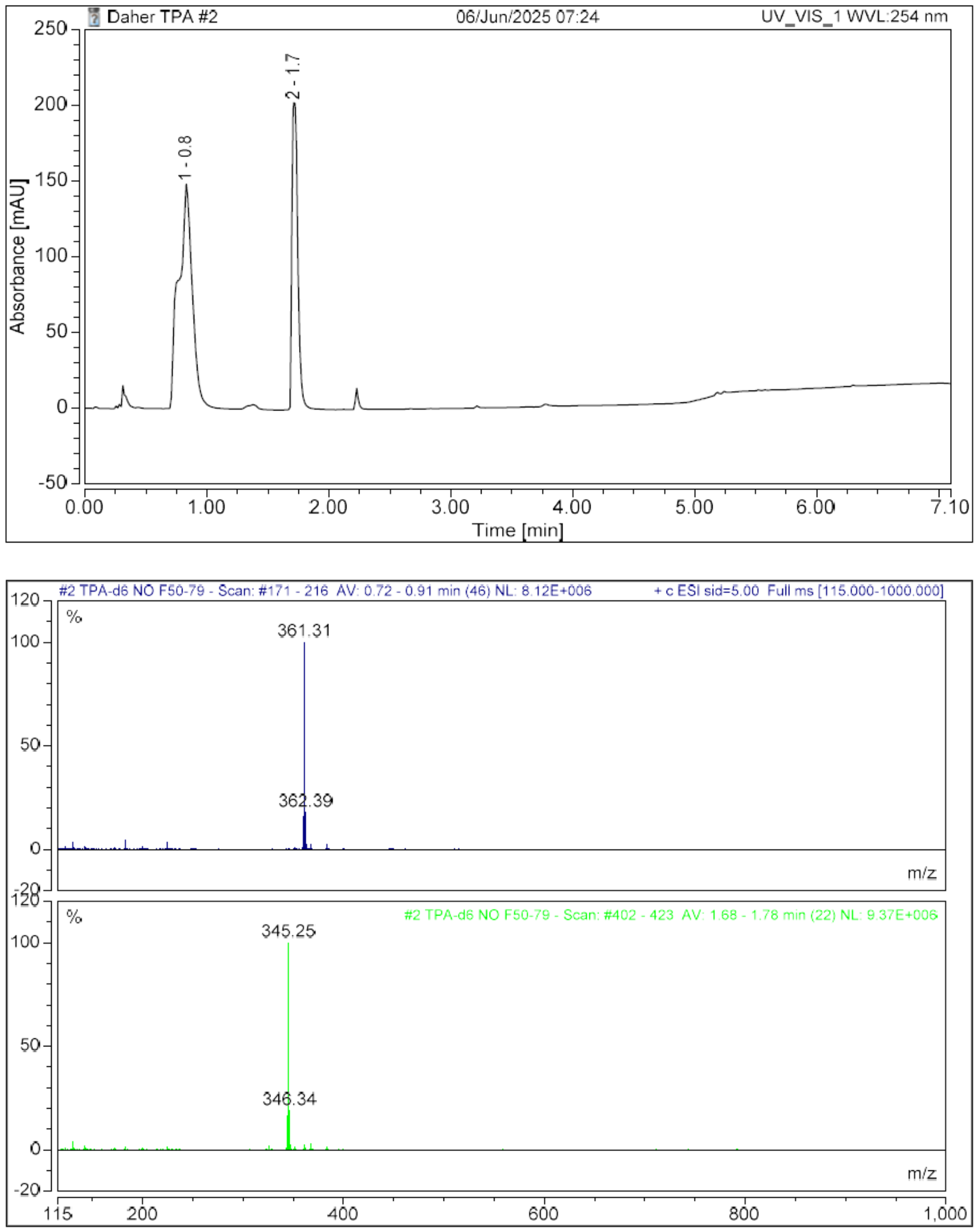

Figure S1. LC-MS analysis of inseparable mixture of N-oxidized **S2.1** $[M+H]^+ = 361$ and **S2.2** $[M+H]^+ = 345$ in methanol. Top: Chromatogram of sample. Conditions: Mobile phase gradient of 0.1% formic acid in acetonitrile/0.1% formic acid in water; column: Phenomenex kinetex C18 1.7 µm, 50 mm; flow: 0.5 mL/min; detector: dual DAD-3000 and MS. Bottom: Mass spectra ESI positive mode.

3. Approach for selective deuteration of TPA

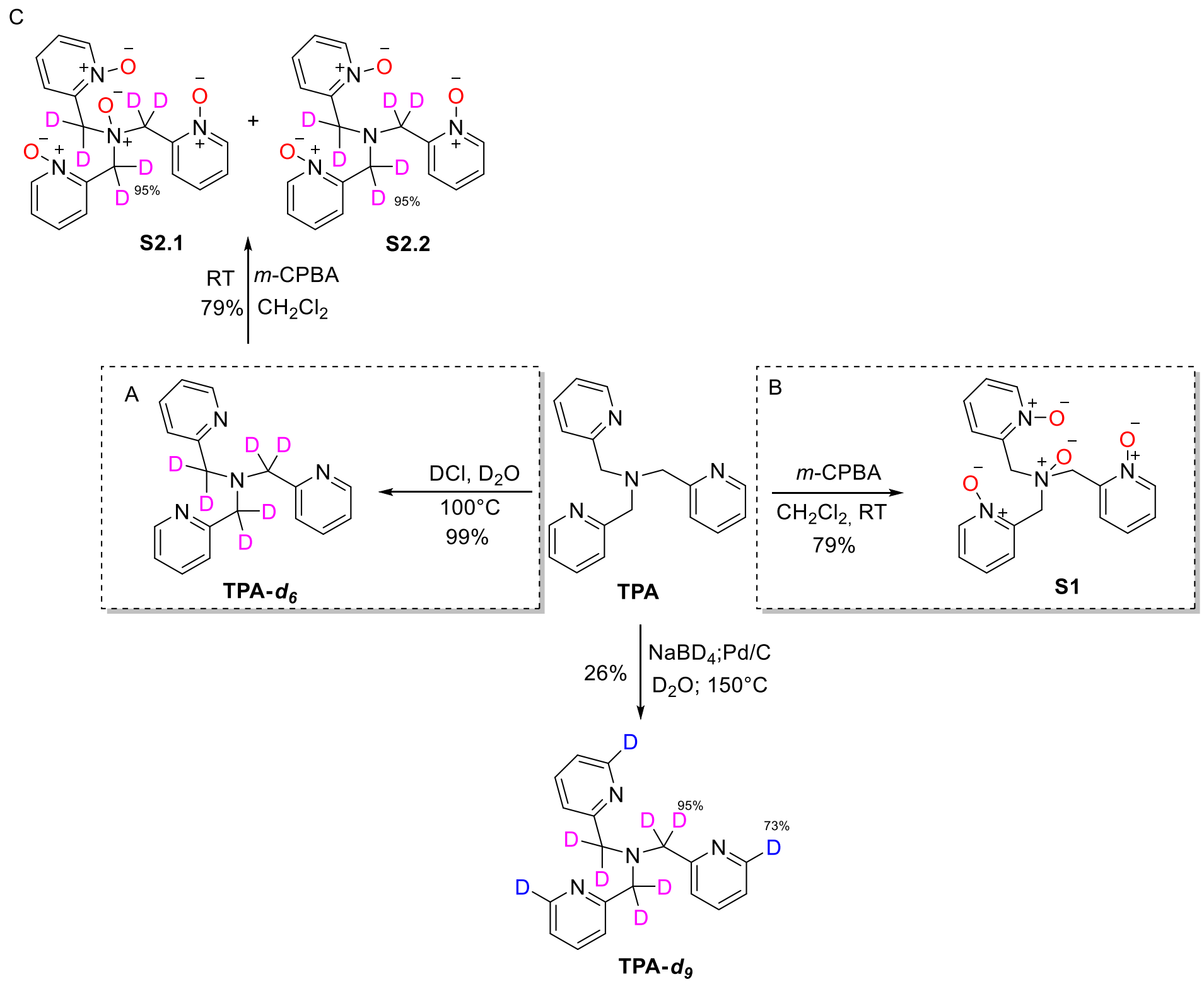

Scheme S1. Synthetic approach for selective deuteration of TPA.

4. Deuteration of TPA *N*-oxide

Table S1. Screening of deuteration conditions over TPA *N*-oxide.

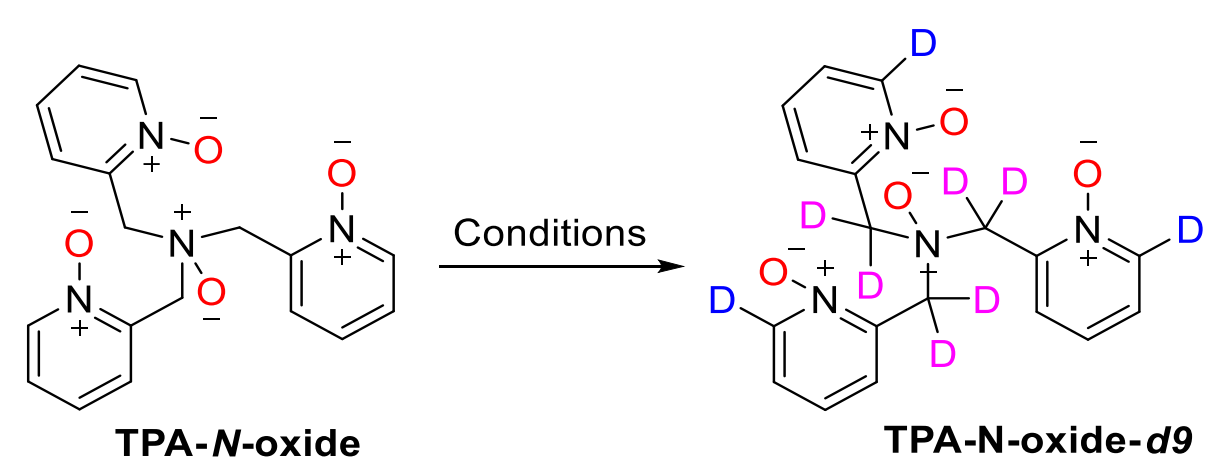

Item	Conditions	Result
1	NaOD / D_2O / 100 °C	Complex mixture of products
2	DCl / D_2O / 100 °C	Complex mixture of products
3	Na_2CO_3 / D_2O / 100 °C	Recovery of starting material
4	DCl / D_2O / 50 °C	Complex mixture, no deuteration
5	NaOD / D_2O / 50 °C	Complex mixture, no deuteration
6	KO^{t}Bu / DMSO-d6 / RT	Complex mixture, no deuteration
7	K_2CO_3 / D_2O / 100 °C	Complex mixture, no deuteration

Deuteration tests were performed as describe on general procedures in section 2. Isolation of selected reactions was carried out by flash column chromatography with mobile phase consisting of CH_2Cl_2:CH_3OH and the isolated fractions are shown below.

5. Quantification of Zn^{2+}.

5.1 Preparation of solutions

I. 25 mM $ZnCl_2$ in 100 mM HEPES buffer 7.0 with citrate.

$ZnCl_2$ solubility in buffer was limited, therefore citric acid was added to increase the solubility. To prepare 25 mM of Zn^{2+} stock solution, $ZnCl_2$ (17 mg, 0.12 mmol) was partially dissolved in 4.6 mL of 100 mM HEPES buffer pH 7.0 followed by the addition of 400 µL of citrate solution 1 M (citric acid 0.9 g, 0.005 mol was dissolved in 100 mM HEPES buffer pH 7.0 followed by the addition of NaOH 1 M solution until pH 7, then the volume was completed adding 100 mM HEPES buffer pH 7.0) for a total volume of 5 mL.

II. 25 mM TPA-d_9 in 100 mM HEPES buffer 7.0.

TPA-d_9 (23 mg, 0.077 mmol) was dissolved in 3 mL of 100 mM HEPES buffer 7.0 with stirring to ensure complete dissolution.

III. 5 mM solutions for zinc quantification in 100 mM HEPES buffer 7.0.

To prepare NMR tubes for zinc sensing, 80 µL of TPA-d_9 stock solution from item 5.1.II were added on the NMR tubes followed by different amounts of Zn^{2+} solution from item 5.1.I in accordance with the table below. After vigorously mixing, the samples were directly measured. Each item was measured in triplicate.

Item	Zn^{2+} eq (mM)	Zn^{2+} V(µL)	HEPES V(µL)
1	0	0	320
2	0.5	8	312
3	1.0	16	304
4	2.5	40	280
5	5.0	80	240

IV. Specificity tests.

Stock solutions of $CaCl_2$ (11 mg in 4 mL of 100 mM HEPES 7.0), CuCl (10 mg in 1 mL of 100 mM HEPES 7.0), NaCl (1.5 mg in 1 mL of 100 mM HEPES 7.0), KCl (1.9 mg in 1 mL of 100 mM HEPES 7.0), $FeCl_3$ (4 mg in 1 mL of 100 mM HEPES 7.0), $FeCl_2$ (3.2 mg in 1 mL of 100 mM HEPES 7.0) and $MgCl_2$ (2.4 mg in 1 mL of 100 mM HEPES 7.0) were prepare. In an NMR

tube, 80 µL of TPA-d_9 stock solution from item 5.1.II were added, followed by the addition of 80 µL of each chloride salt plus 240 µL of 100 mM HEPES buffer 7.0 to have a total of 400 µL solution. Then ^2H NMR spectra were recorded.

5.2 Equation used for quantification of Zn^{2+} concentration

The quantification of Zn^{2+} concentration was calculated from the peak areas of the ^2H signal of free TPA-d_9 and the Zn-TPA-d_9 complex, based on the assumption that TPA reacts quantitatively with Zn^{2+}.[5] Knowing the TPA-d_9 concentration enables the quantification of Zn^{2+} concentration as follows:

$$[Zn^{2+}] = \frac{A_1}{A_2+A_1} \times [TPA - d_9] \qquad \text{Supplementary Eq. 1}$$

A_1 = integral corresponding to TPA-d_9-Zn^{2+} complex at 8.80 ppm (for pyridine deuterium signal) or 4.23 ppm (for aliphatic deuterium signal)

A_2 = integral corresponding to TPA-d_9 at 8.38 ppm (for pyridine deuterium signal) or 3.82 ppm (for aliphatic deuterium signal)

[TPA-d_9] = total concentration of TPA-d_9 in NMR tube.

Integration of each measured point was integrated as follows: base line correction (polynomial fit), peak peaking and peak fitting/deconvolution adjusting the shape type with Lorentzian-Gaussian and a width of 13.7-14.1 Hz for unbound TPA-d_9 signals and 22-25 Hz for Zn-TPA-d_9 bound signals. For 0.5 mM a width of 20-22.5 Hz was used. Additionally, the position of constraint was set to 10% for unbound and 5% for bound.

5.3 Detection (LOD) and quantification (LOQ) limits

From the titration curve obtained in section 5.2, a data analysis of the regression curve was performed in Microsoft Excel. The Anova output was used to calculate the limits as follows:

LOD = 3.3 x σ / m

LOQ = 10 x σ / m

Where, σ = standard deviation of the response and m = calibration curve´s slope

[5] Suh, E.H., Park, J.M., Lumata, L. *et al. Hyperpolarized ^{15}N-labeled, deuterated tris(2-pyridylmethyl)amine as an MRI sensor of freely available Zn^{2+}. Commun Chem.* **2020** 3, 185.

6. Relaxation time of deuterium measurement

55 mM solutions in 100 mM HEPES buffer 7.0 of TPA-*d*$_9$ and Zn-TPA-*d*$_9$ complex were measured to determine longitudinal relaxation time (T_1) via the inversion recovery sequence.

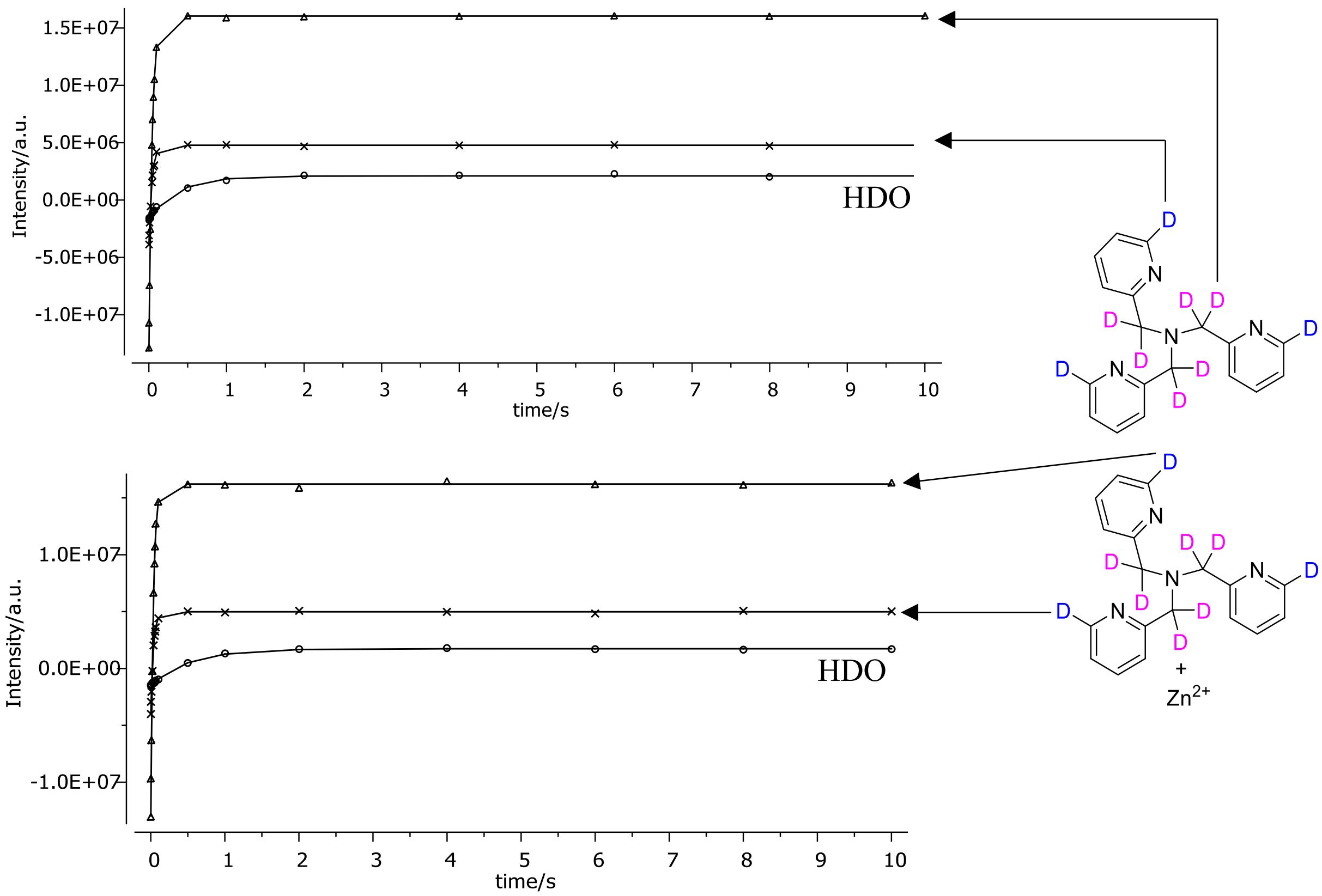

Figure S2. Longitudinal relaxation time (T_1) measurement via inversion recovery of: top: TPA-*d*$_9$ in 100mM HEPES buffer 7.0 at 298 K and bottom: Zn-TPA-*d*$_9$ complex in 100 mM HEPES buffer 7.0 at 298 K.

7. Selectivity test results on TPA-d_9

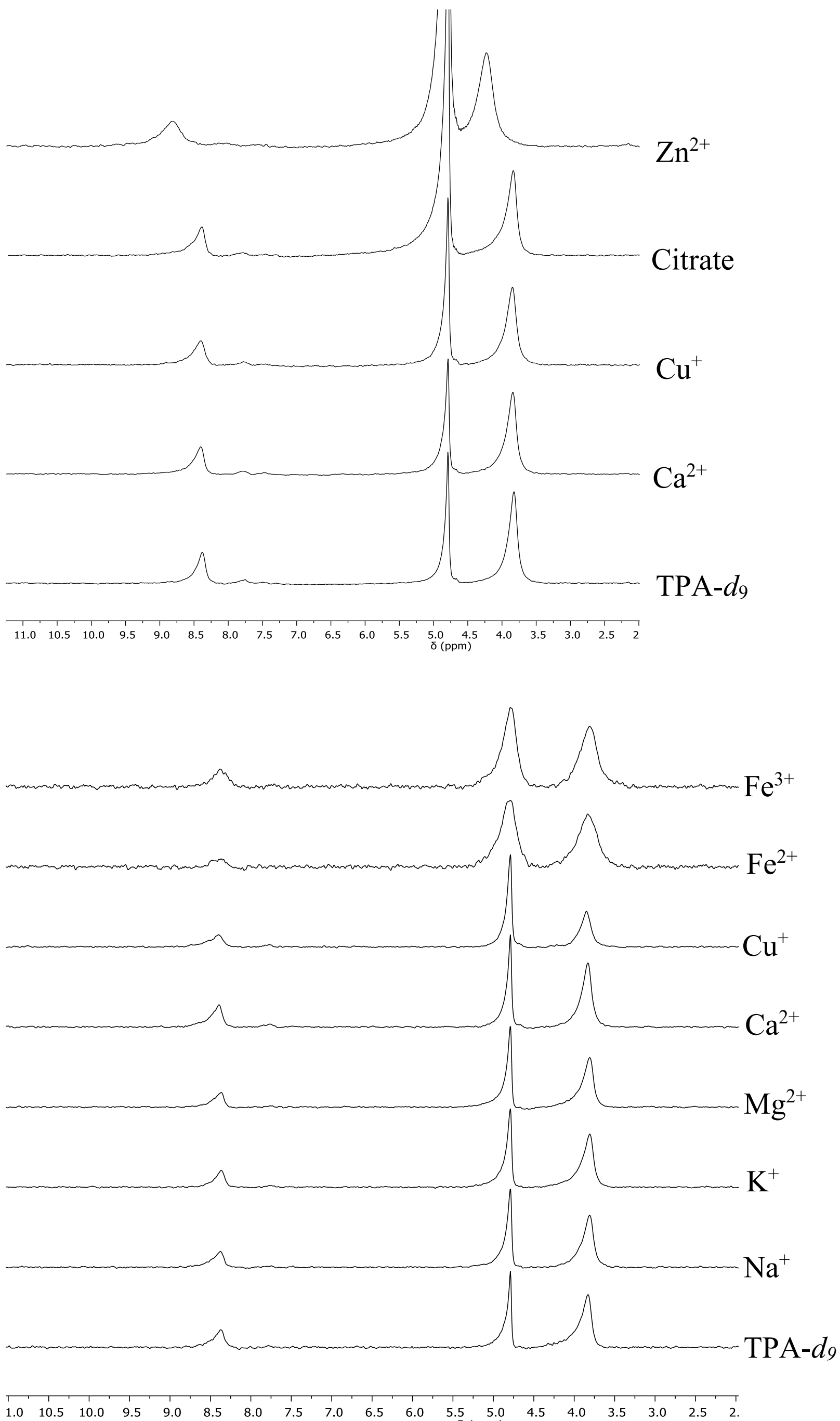

Figure S3. Top: ^{2}H NMR (600 MHz) of TPA-d_9 (5 mM) in 100 mM HEPES buffer 7.0 at 298 K. From top to bottom: $ZnCl_2$ (5 mM); citrate (5 mM); CuCl (5 mM); $CaCl_2$ (5 mM). Bottom: ^{2}H NMR (700 MHz) of TPA-d_9 (5 mM) in 100 mM HEPES buffer 7.4 at 298 K. From top to bottom: $FeCl_3$ (5 mM); $FeCl_2$ (5 mM); CuCl (5 mM); $CaCl_2$ (5 mM); $MgCl_2$ (5 mM); KCl (5 mM); NaCl (5 mM), TPA-d_9 (5 mM).

8. Products identified with $NaBD_4$ deuteration

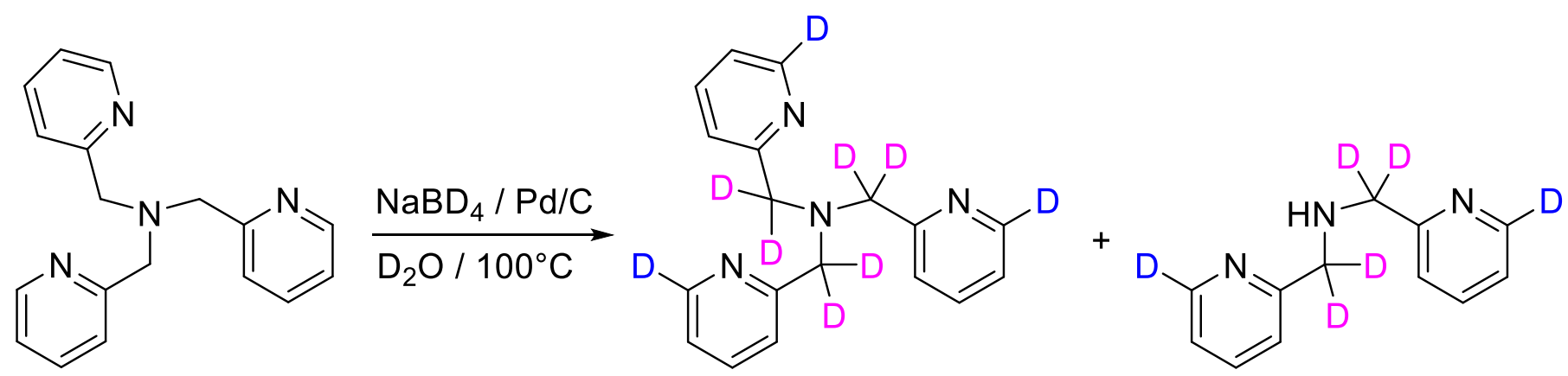

TPA **TPA-*d*₉** **S-3**

When TPA is subject to deuteration under $NaBD_4$ conditions, cleavage products were spotted. After careful isolation two fractions were obtained; the first eluted spot corresponds to the desired product **TPA-*d*$_9$** and the second one corresponds to an inseparable mixture of **TPA-*d*$_9$** and **S3**, product of central amino N-C cleavage with a 0.4 : 0.6 ratio as was identified by LC-MS analysis.

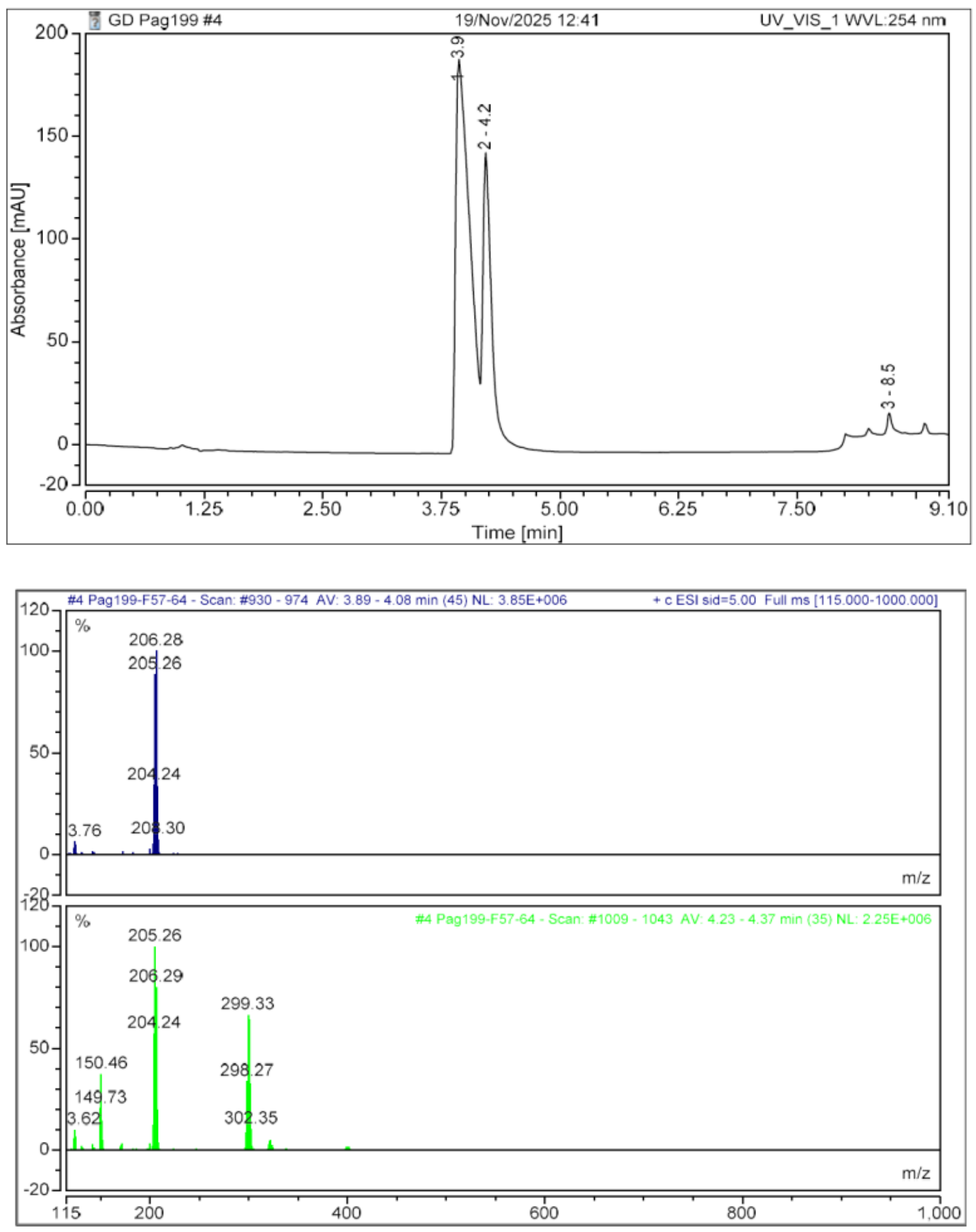

Figure S4. LC-MS analysis of inseparable mixture of **TPA-*d*$_9$** $[M+H]^+ = 300$ and **S3** $[M+H]^+ = 206$ in methanol. Top: Chromatogram of sample, conditions: Mobile phase gradient of 0.1% formic acid in acetonitrile/0.1% formic acid in water; column: Phenomenex kinetex C18 1.7 µm, 50 mm; flow: 0.5 mL/min; detector: dual DAD-3000 and MS. Bottom: Mass spectra ESI positive mode.

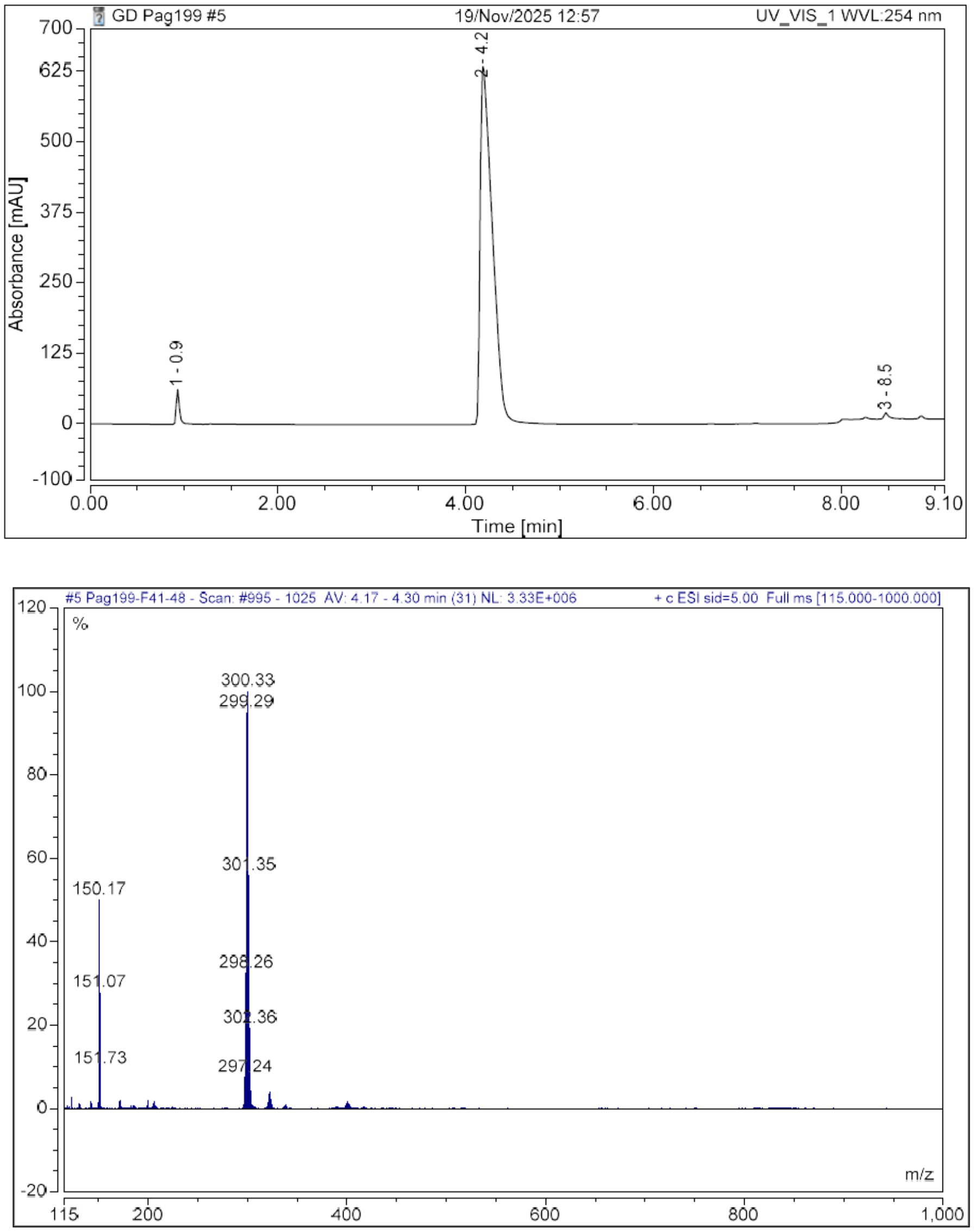

Figure S5. LC-MS analysis of **TPA-*d*$_9$** $[M+H]^+ = 300$ in methanol. Top: Chromatogram of sample, conditions: Mobile phase gradient of 0.1% formic acid in acetonitrile/0.1% formic acid in water; column: Phenomenex kinetex C18 1.7 µm, 50 mm; flow: 0.5 mL/min; detector: dual DAD-3000 and MS. Bottom: Mass spectra ESI positive mode.

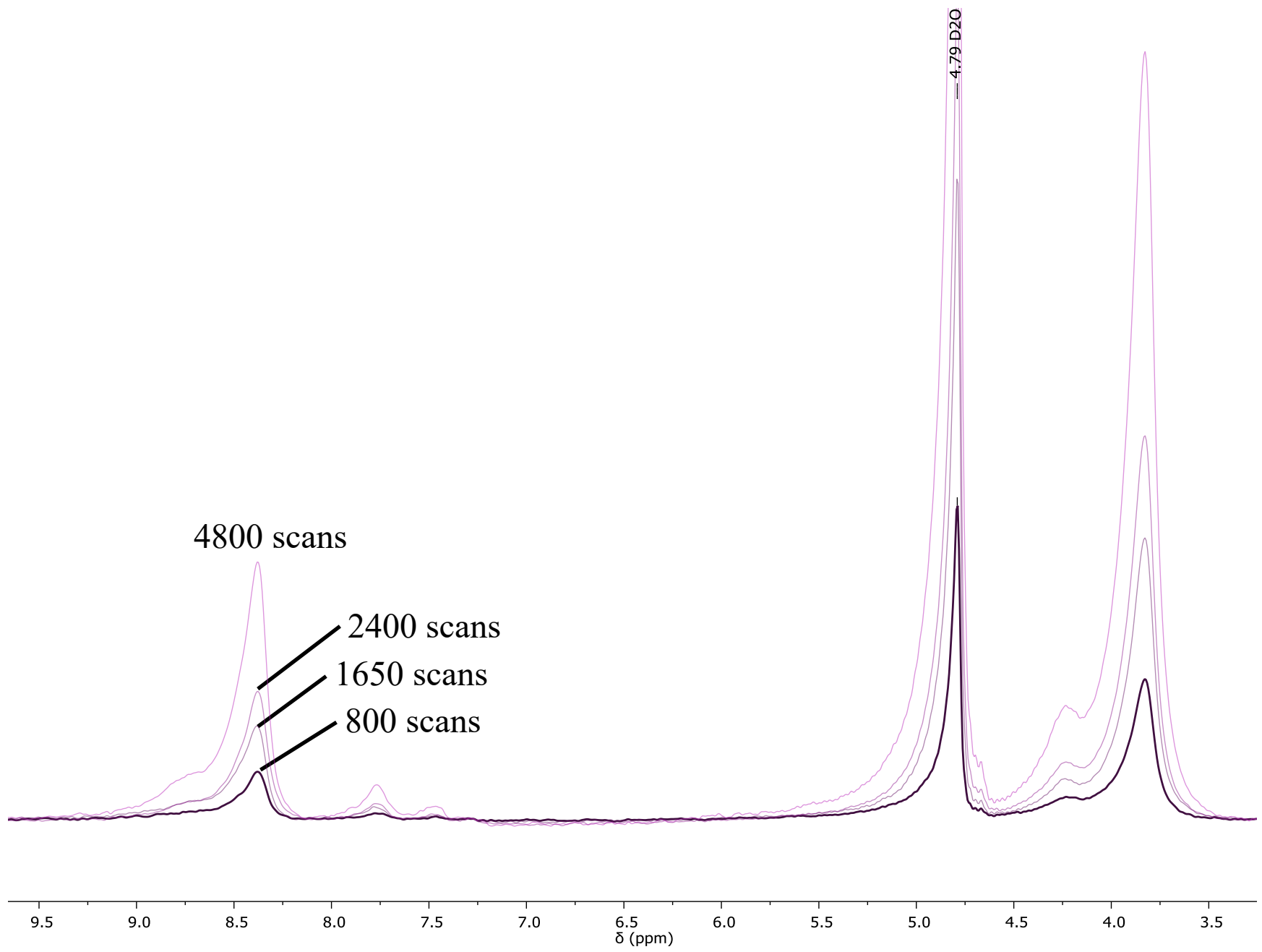

Figure S6. ^{2}H NMR (600 MHz) of TPA-d_9 (5 mM) with $ZnCl_2$ (0.5 mM) in 100 mM HEPES buffer 7.0 at 298 K at various number of scans. To provide information regarding quantification accuracy when measuring Zn^{2+} with pyridine deuteron, 800 scans is sufficient but longer acquisition times proved to yield better integration windows.

9. Cells experiments

a. Cell culture

Cervical carcinoma (HeLa) cells were cultured in Dulbecco's Modified Eagle Medium (DMEM, high glucose) supplemented with 10% fetal bovine serum (FBS) and 1% penicillin/streptomycin at 37 °C in a humidified 5% CO_2 atmosphere. Cells were routinely maintained in T175 flasks and passaged at 90–100% confluence using 0.25% trypsin/EDTA.

b. Sample preparation for NMR Spectroscopy

For NMR experiments, HeLa cells were cultured to a 90% confluence. Approximately two T175 flasks were used per experiment, corresponding to approximately 60×10^6 cells.

Cells were washed once with DPBS and mechanically detached by addition of 10 mL DPBS buffer per flask followed by gentle scraping. Trypsin was not used during harvesting to preserve membrane integrity.

Cells from all flasks belonging to the same experiment were pooled into 50 mL conical tubes and centrifuged at 3000 rpm for 3 min at room temperature.

Following centrifugation, the supernatant was carefully removed, and the dense cell pellet was resuspended in approximately 150 µL of 100 mM HEPES, 100 mM NaCl, and 5 mM glucose buffer (pH 7.4), yielding a final slurry volume of approximately 320 µL.

The concentrated cell suspension (300 µL) was transferred into 5 mm Shigemi NMR tubes while avoiding introduction of air bubbles.

c. Results

To translate the probes performance to a cell environment, we first evaluated the feasibility of using a 600 MHz NMR spectrometer. When the probe was dissolved in buffer and added to the cell suspension, no signal was observed, neither with spike of Zn-pyrithione or without (item 1 and 2, figure S7). We therefore decided to move to a higher magnetic field (700 MHz). When the probe was dissolved in buffer with 0.5 mM of $ZnCl_2$(item 3) or dissolved in DMSO in the same conditions(item 4) strong signals were observed after only 400 scans (10 min acquisition). When moving on to HeLa cells, almost no signal is observed in the measurement buffer (item 5 compared to item 3 or 4). Addition of the probe dissolved in DMSO renders a signal in the cell suspension (item 6), indicating possible membrane permeation and interaction with larger proteins. This interaction causes the signal to broaden and thus disappear in the noise. However, it can be recovered by addition of small amounts of DMSO (here in form of dissolving the probe in DMSO before addition to the cells).[6] To achieve a better SNR for the zinc sensing experiments, the number of scans was increased after addition of 0.6 mM of $ZnCl_2$ (items 7 and 8). The addition of increasing amounts of $ZnCl_2$ (items 8 - 11) results in a decrease and broadening of the TPA-d_9 signal, which leads to the signal almost completely disappearing at 1.8 mM $ZnCl_2$ (item 11). Quantification, at this point, is not achievable since, most likely, the Zn-probe complex is buried under the residual HDO peak.

[6] Tunçer S., Gurbanov R., Sheraj I., Solel E., Esenturk O., Banerjee S. *Low dose dimethyl sulfoxide driven gross molecular changes have the potential to interfere with various cellular processes*. *Sci Rep*. **2018**, 8(1):14828.

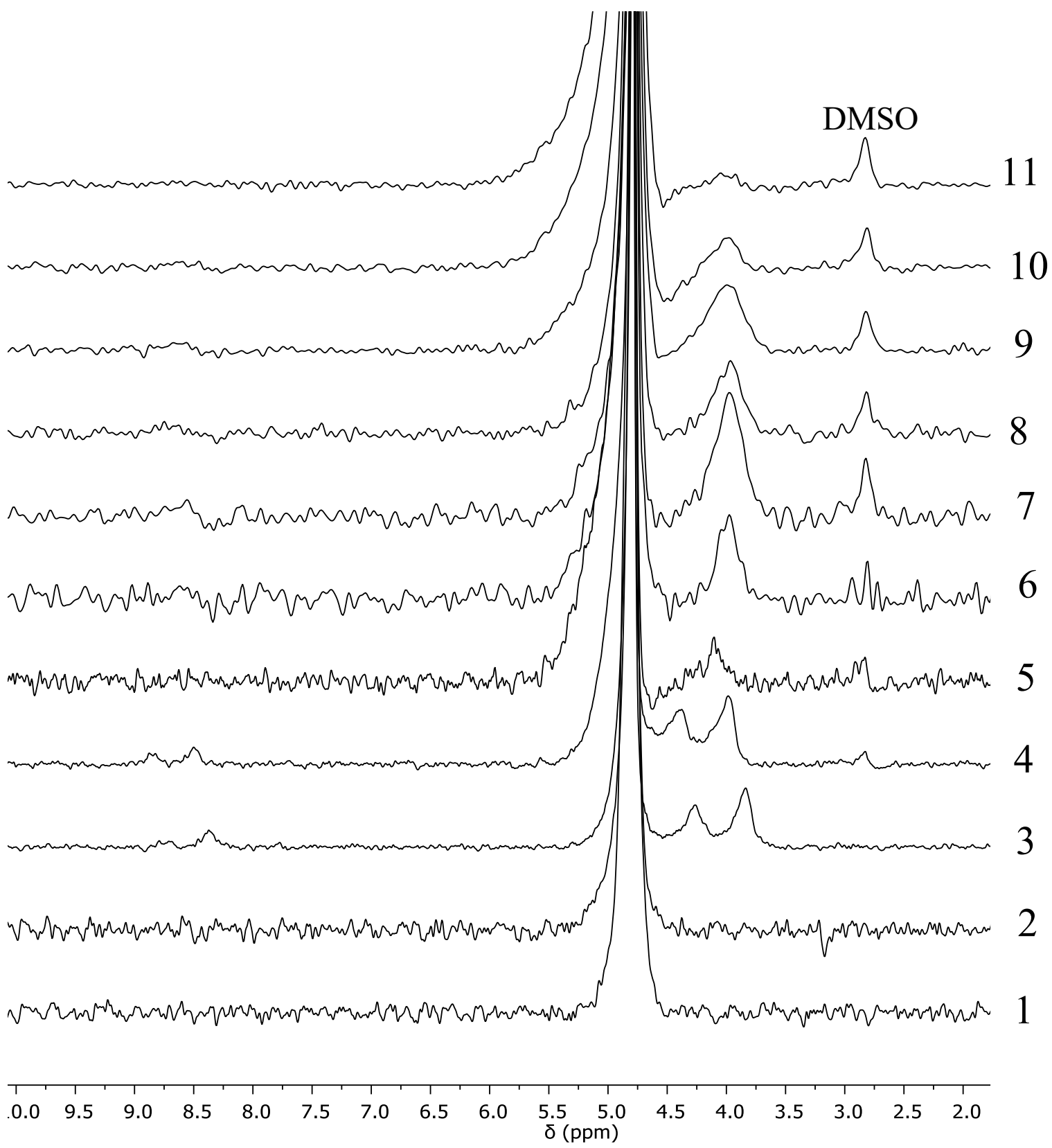

1) HeLa cells + 1 mM TPA-d_9 in 100 mM HEPES buffer 7.4 + 100 mM NaCl + 5 mM glucose (600 MHz, 800 scans, 300 µL)
2) 1 mM TPA-d_9 + spike 0.3 mM Zn-pyrithione in 100 mM HEPES buffer 7.4 + 100 mM NaCl + 5 mM glucose (600 MHz, 800 scans, 300 µL)
3) 1 mM TPA-d_9 + 0.5 mM $ZnCl_2$ in 100 mM HEPES buffer 7.4 + 100 mM NaCl + 5 mM glucose (700 MHz, 400 scans, 400 µL)
4) 1 mM TPA-d_9 (in DMSO) + 0.5 mM $ZnCl_2$ in 100 mM HEPES buffer 7.4 + 100 mM NaCl + 5 mM glucose (700 MHz, 400 scans, 400 µL)
5) HeLa cells + 1 mM TPA-d_9 in 100 mM HEPES buffer 7.4 + 100 mM NaCl + 5 mM glucose (700 MHz, 400 scans, 300 µL)
6) HeLa cells + 1 mM TPA-d_9 (in DMSO) + 100 µL of 100 mM HEPES buffer 7.4 + 100 mM NaCl + 5 mM glucose (700 MHz, 400 scans, 400 µL)
7) HeLa cells + 1 mM TPA-d_9 (in DMSO) + 0.6 mM $ZnCl_2$ in 100 mM HEPES buffer 7.4 + 100 mM NaCl + 5 mM glucose (700 MHz, 400 scans, 400 µL)
8) HeLa cells + 1 mM TPA-d_9 (in DMSO) + 0.6 mM $ZnCl_2$ in 100 mM HEPES buffer 7.4 + 100 mM NaCl + 5 mM glucose (700 MHz, 1200 scans, 400 µL)
9) HeLa cells + 1 mM TPA-d_9 (in DMSO) + 1.1 mM $ZnCl_2$ in 100 mM HEPES buffer 7.4 + 100 mM NaCl + 5 mM glucose (700 MHz, 1200 scans, 400 µL)
10) HeLa cells + 1 mM TPA-d_9 (in DMSO) + 1.4 mM $ZnCl_2$ in 100 mM HEPES buffer 7.4 + 100 mM NaCl + 5 mM glucose (700 MHz, 1200 scans, 400 µL)
11) HeLa cells + 1 mM TPA-d_9 (in DMSO) + 1.8 mM $ZnCl_2$ in 100 mM HEPES buffer 7.4 + 100 mM NaCl + 5 mM glucose (700 MHz, 1200 scans, 400 µL)

Figure S7. ^{2}H NMR (700 MHz, 310 K) of HeLa cells with TPA-d_9 1 mM. Conditions described in bottom of the NMR spectra.

10. ^{1}H, ^{2}H and ^{13}C NMR of new compounds

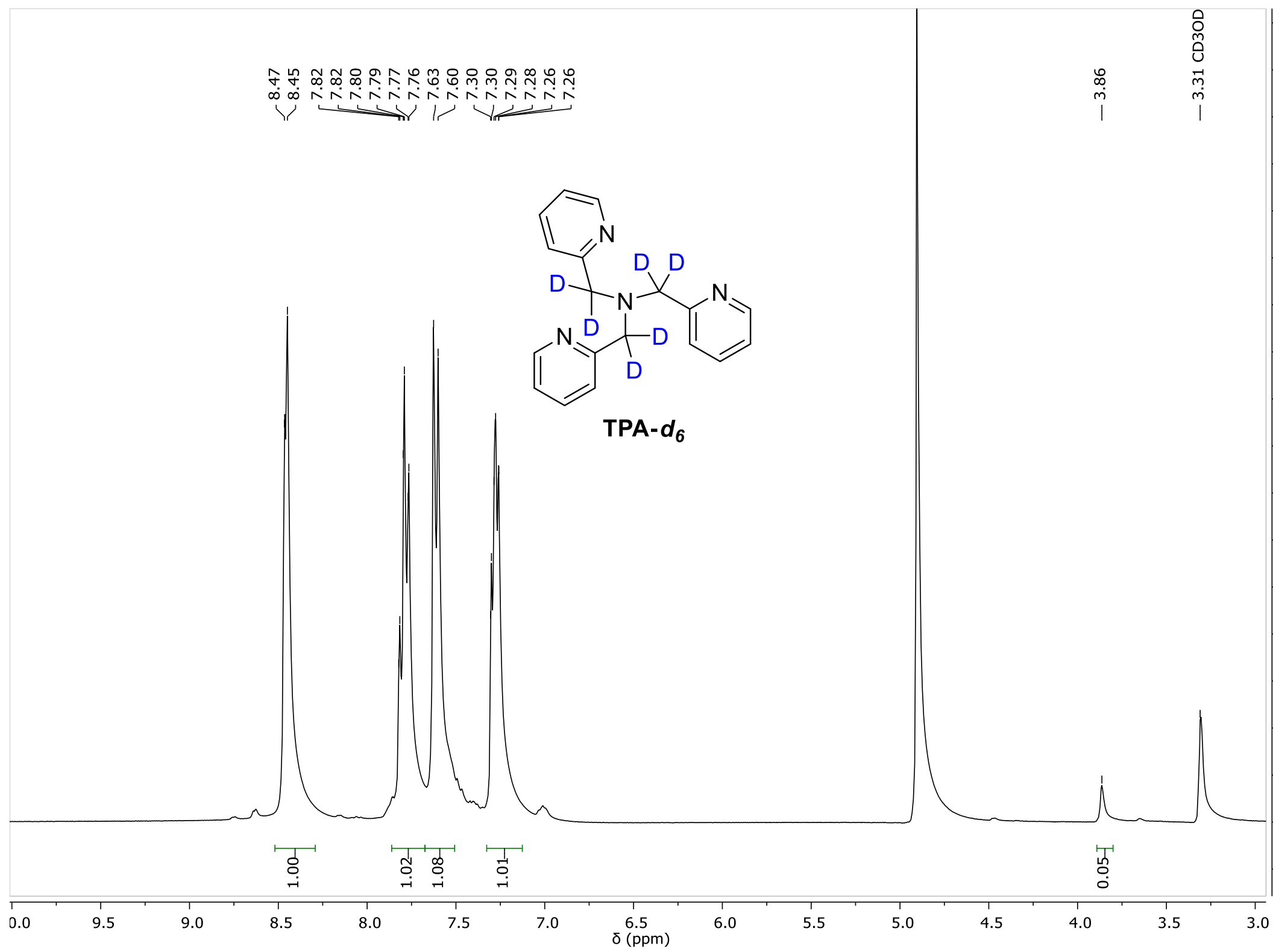

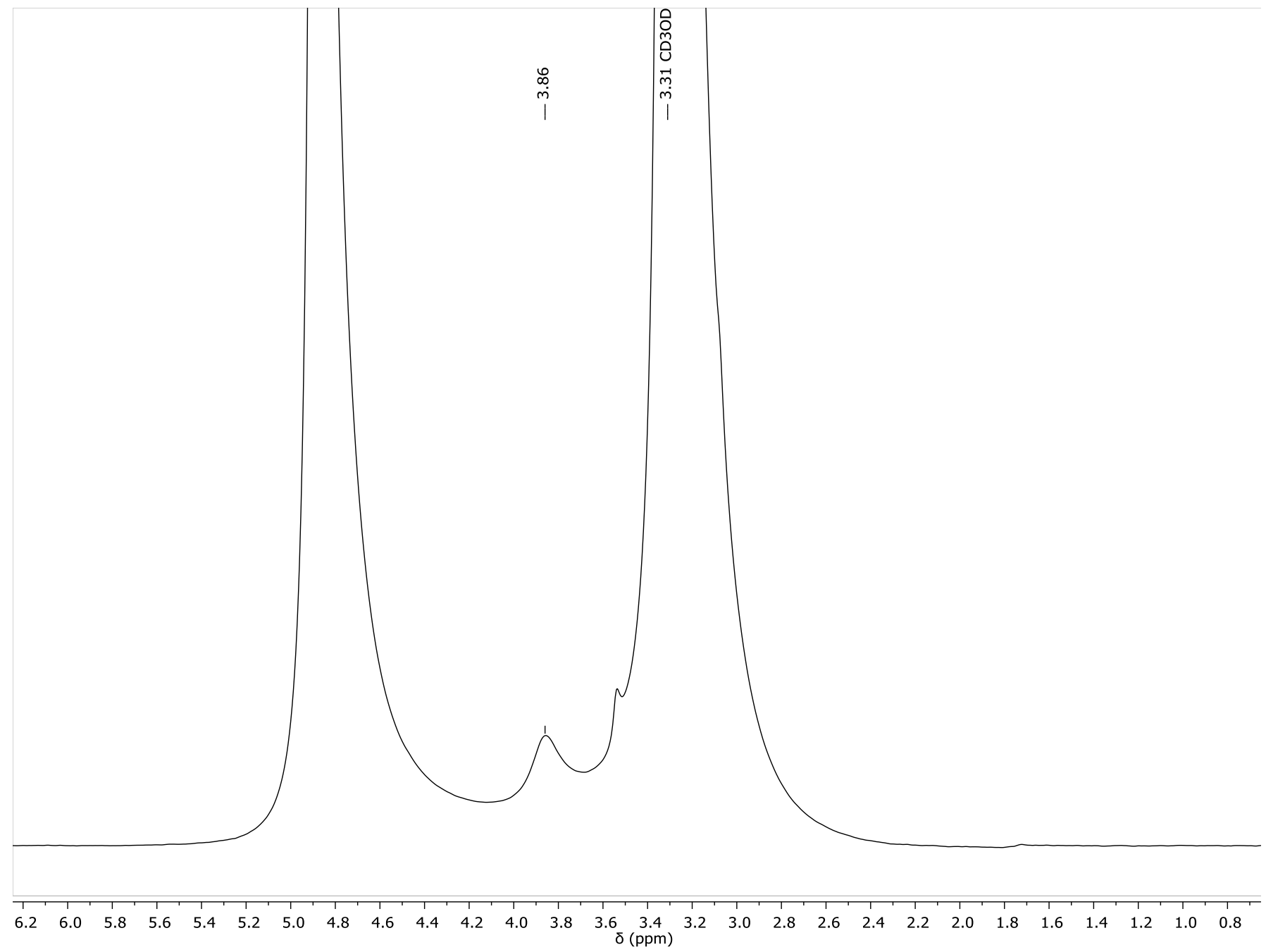

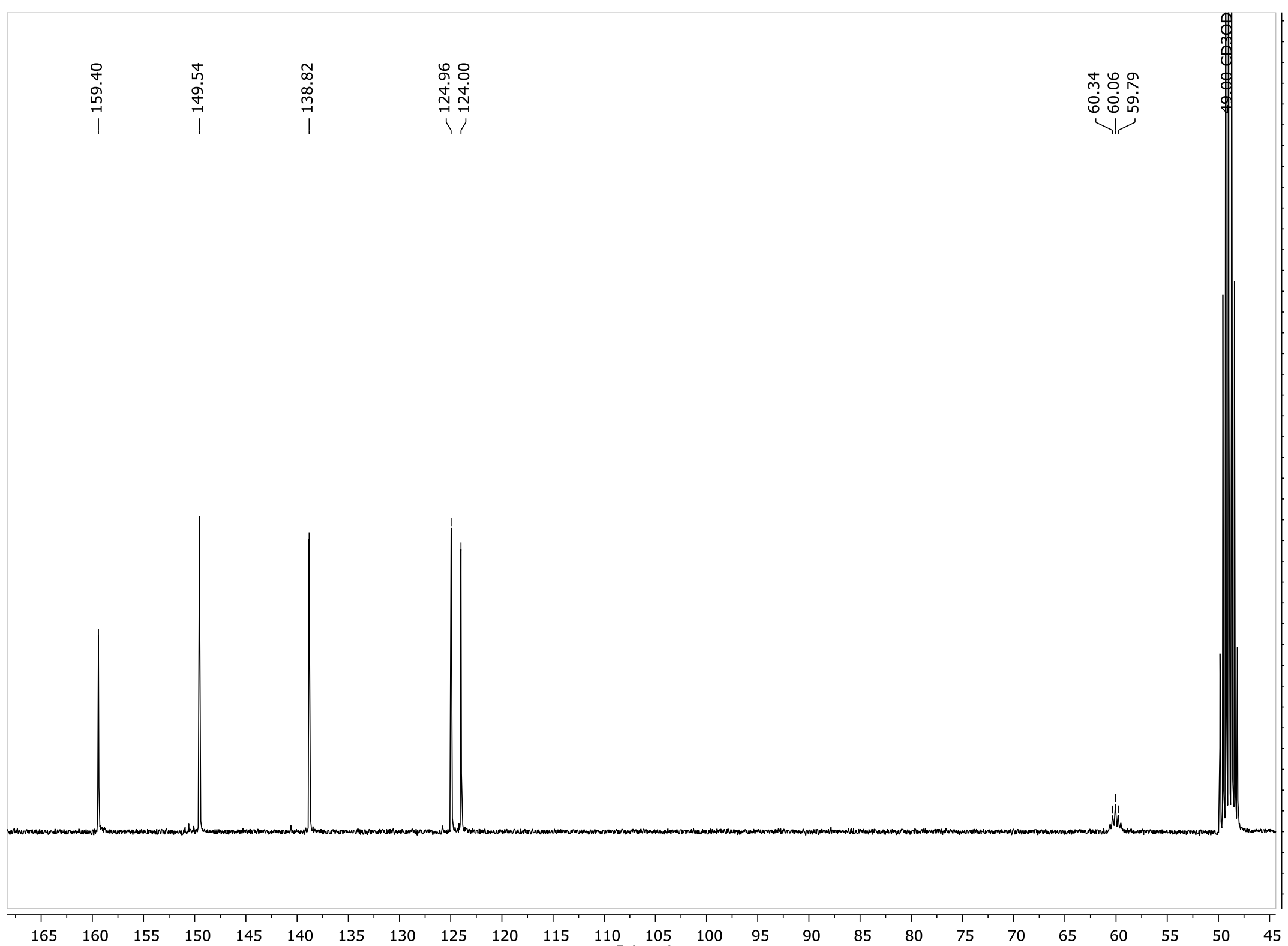

Figure S8. ^{1}H, ^{2}H and ^{13}C NMR (300 MHz) of TPA-d_6 in methanol-d_4 at 298 K.

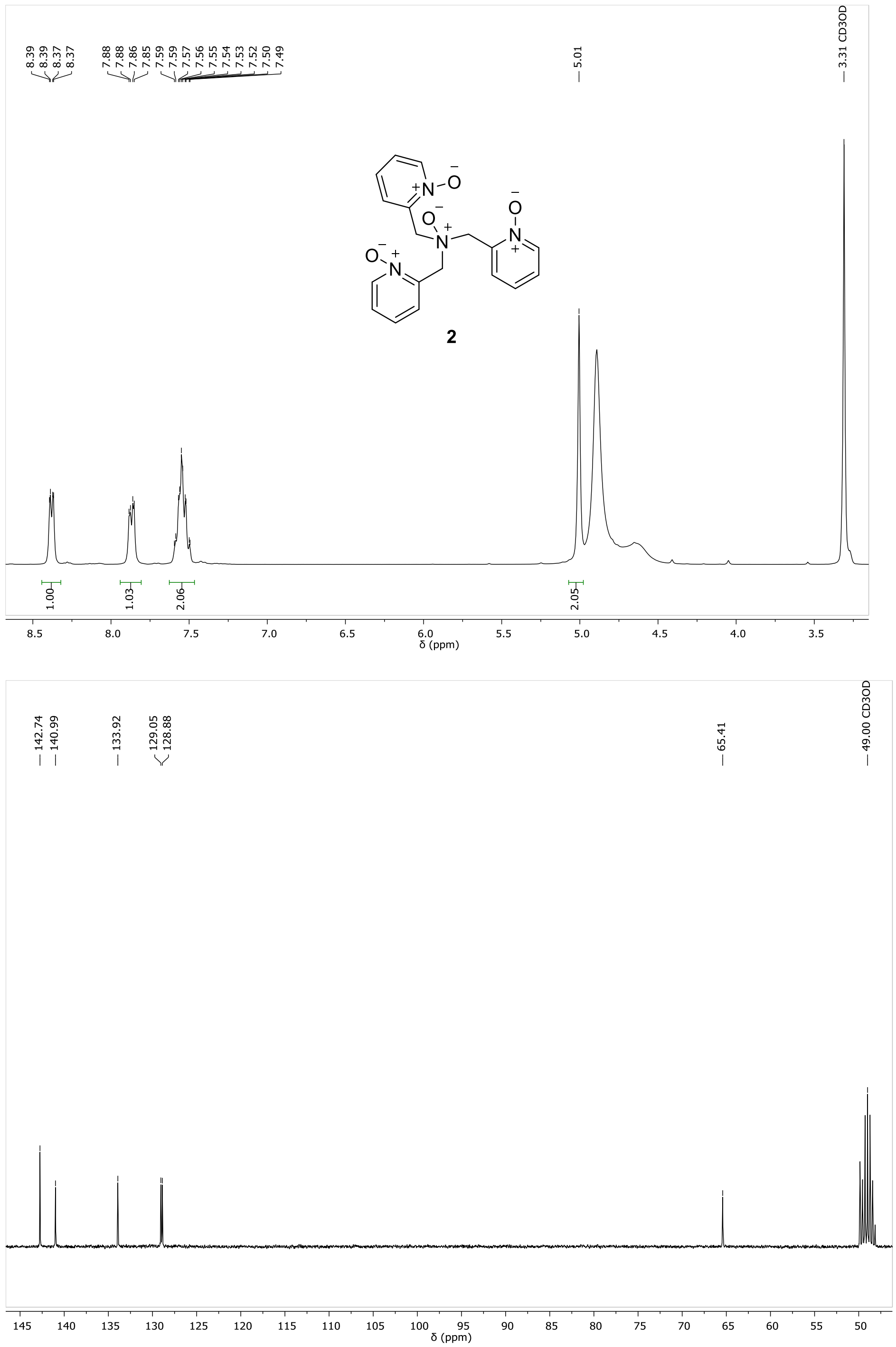

Figure S9. ^{1}H and ^{13}C NMR (300 MHz) of TPA-N-oxide **2** in methanol-d_4 at 298 K.

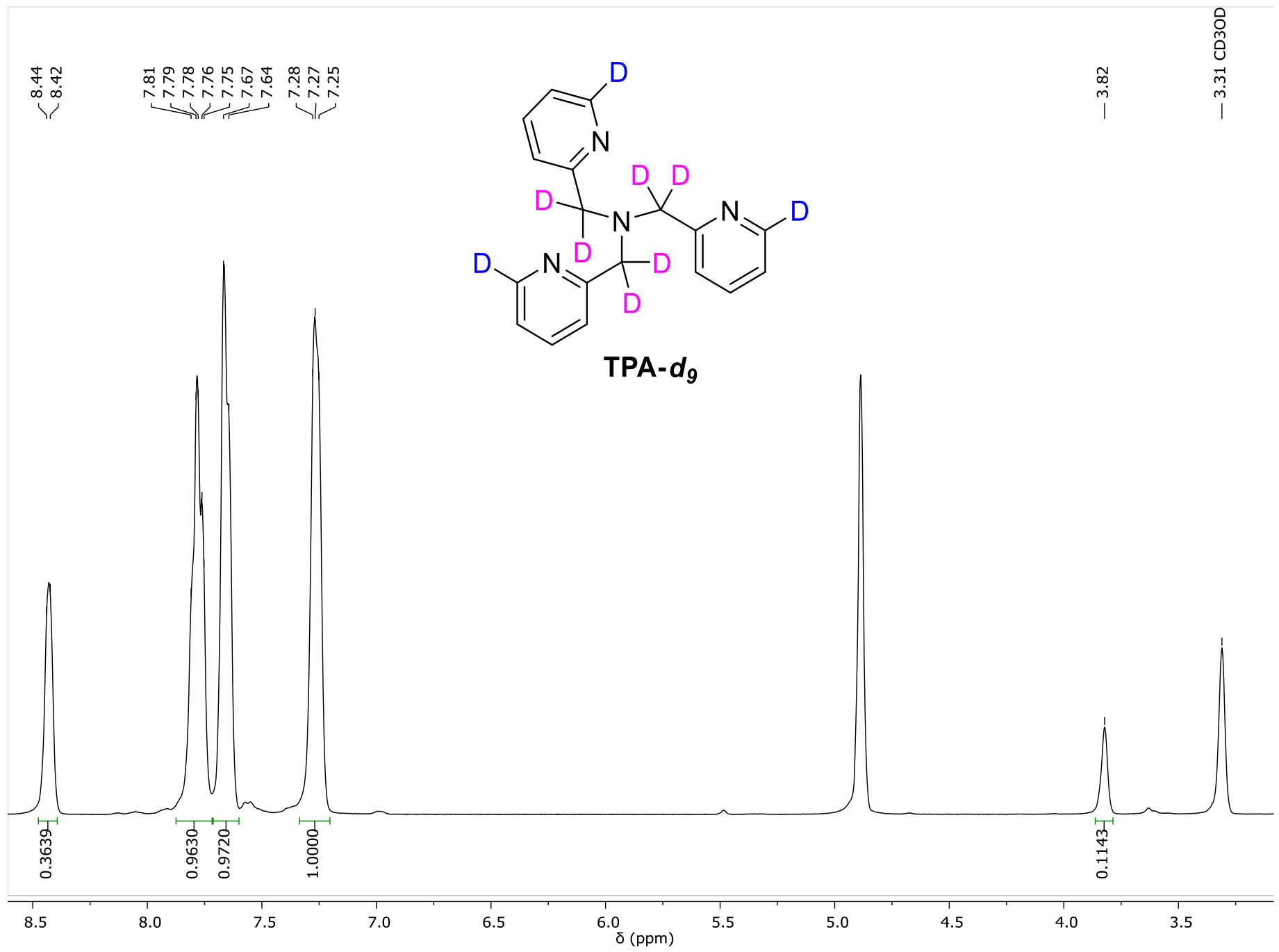
8.44
8.42
7.81
7.79
7.78
7.76
7.75
7.67
7.64
7.28
7.27
7.25
3.82
3.31 CD3OD
TPA-d_9
0.3639
0.9630
0.9720
1.0000
0.1143
δ (ppm)

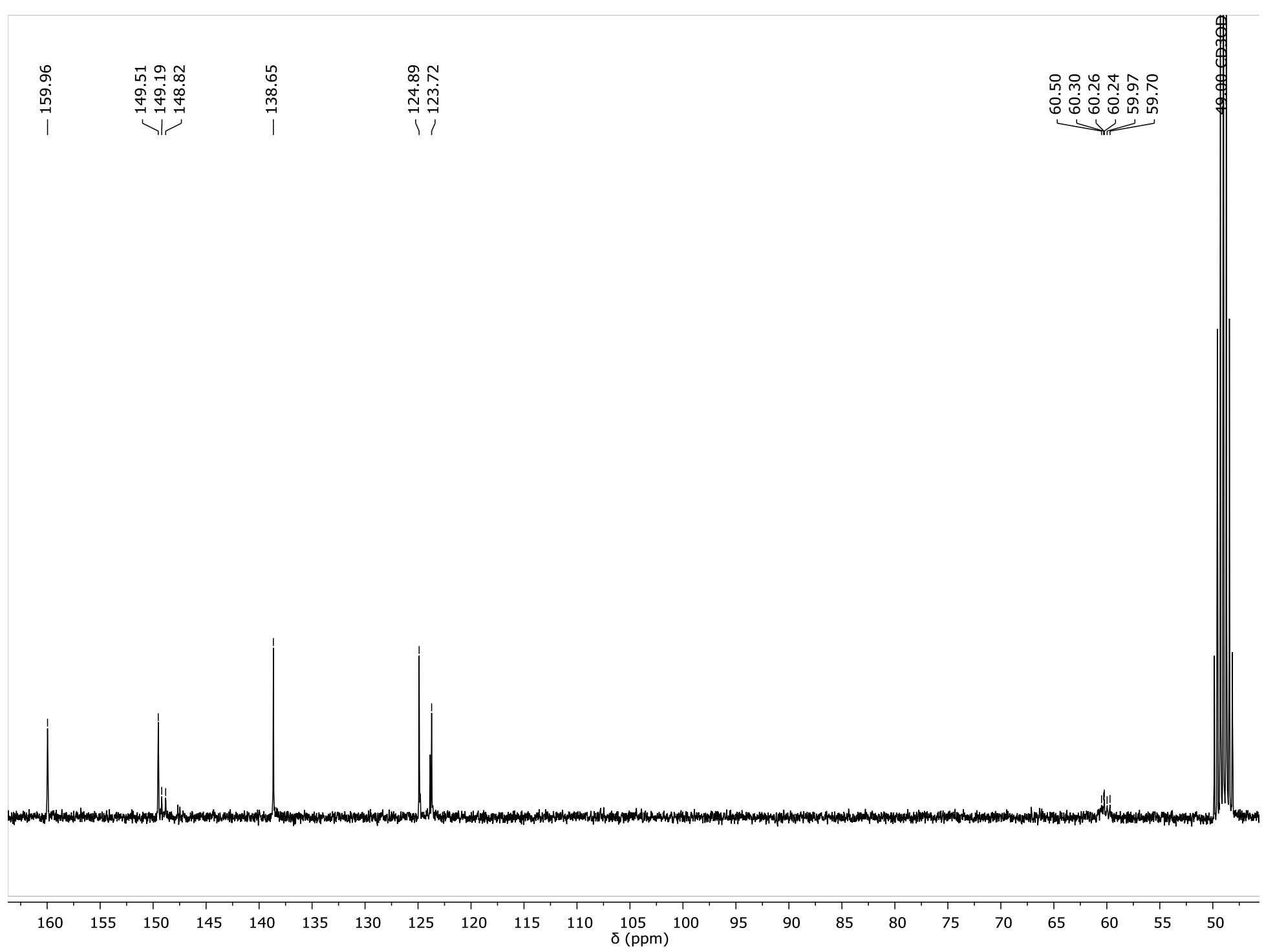
159.96
149.51
149.19
148.82
138.65
124.89
123.72
60.50
60.30
60.26
60.24
59.97
59.70
49.00 CD3OD
δ (ppm)

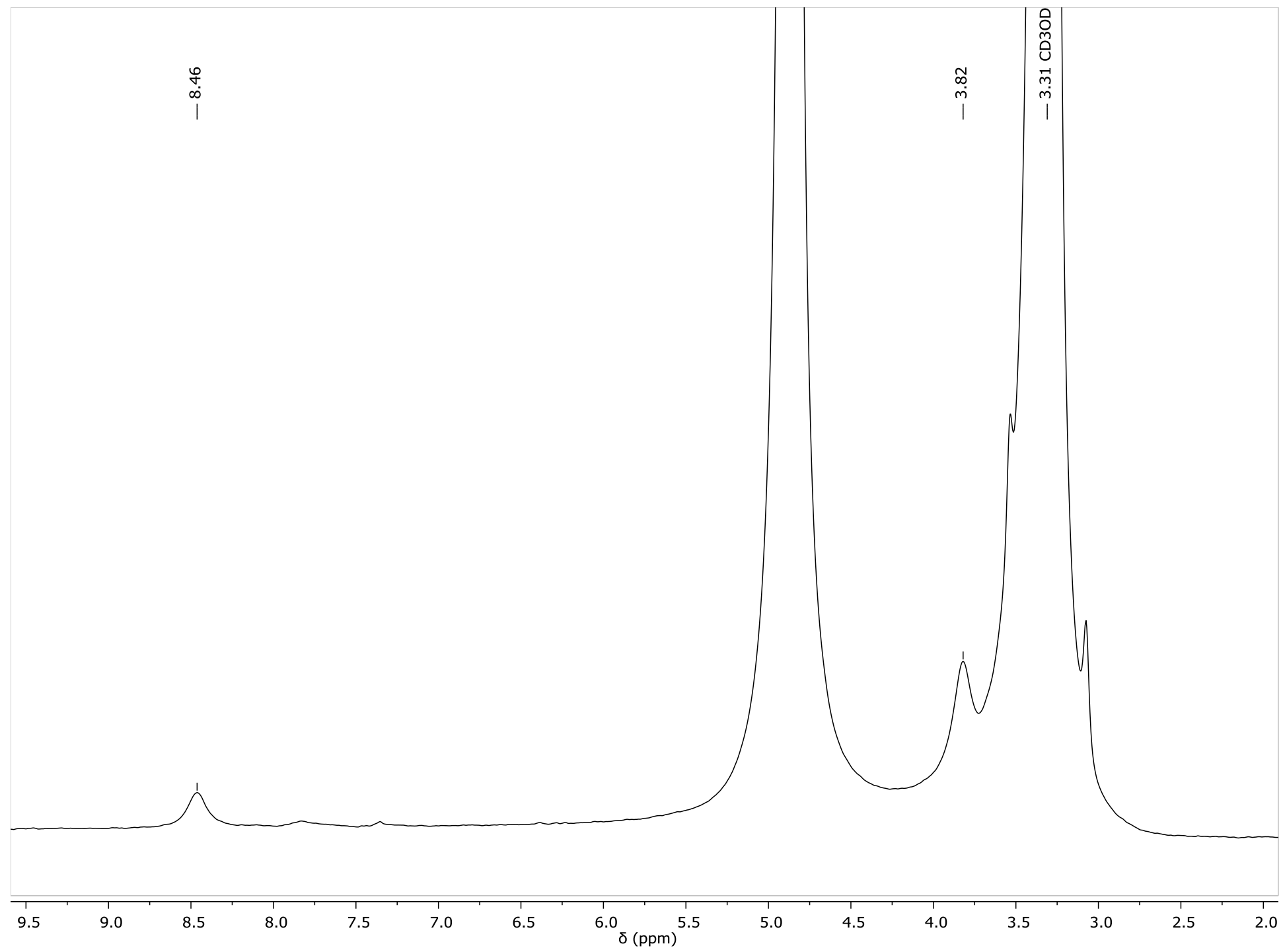

Figure S10. ^{1}H and ^{13}C NMR (300 MHz) of TPA-d_9 in methanol-d_4 at 298 K.

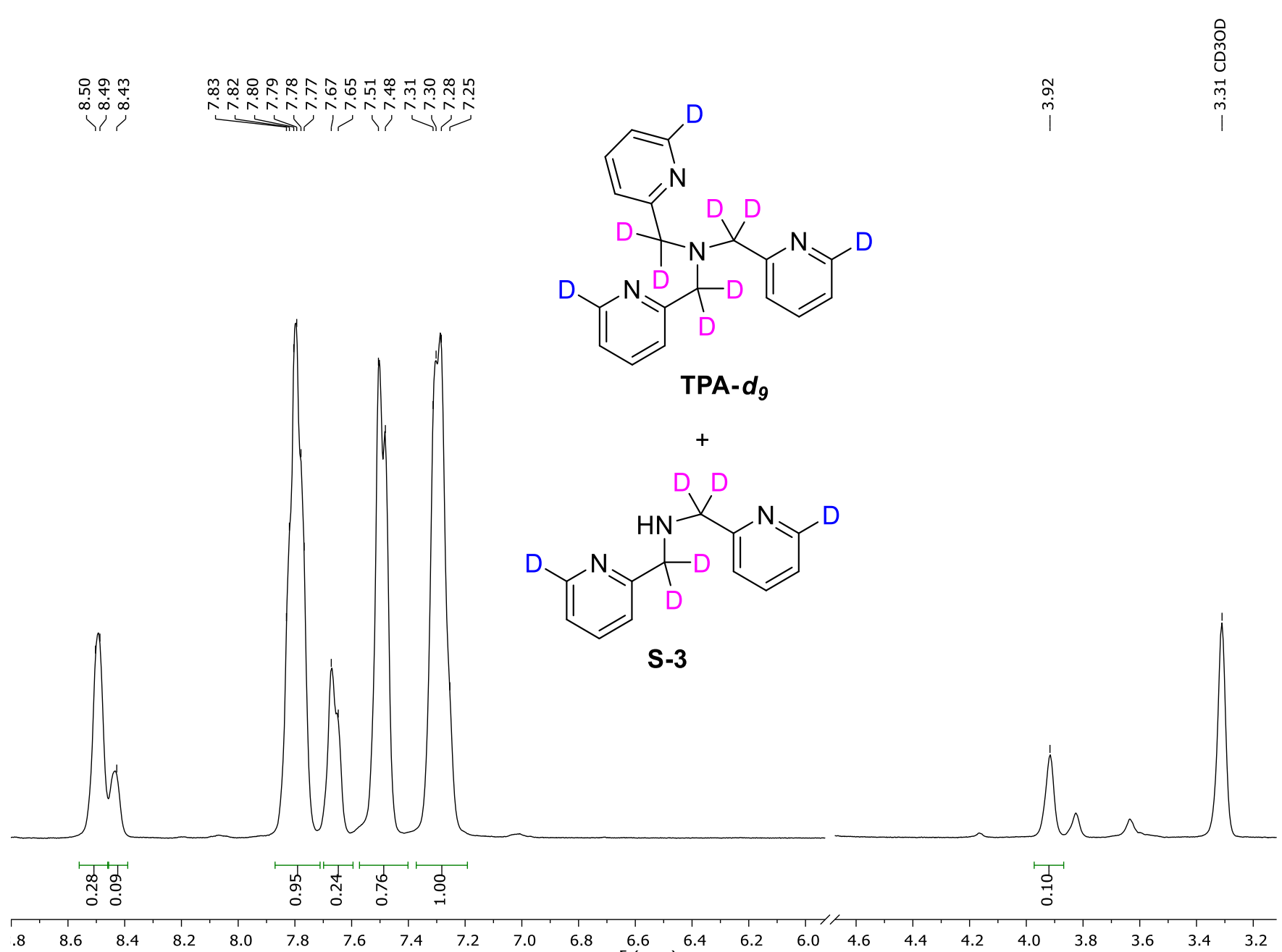

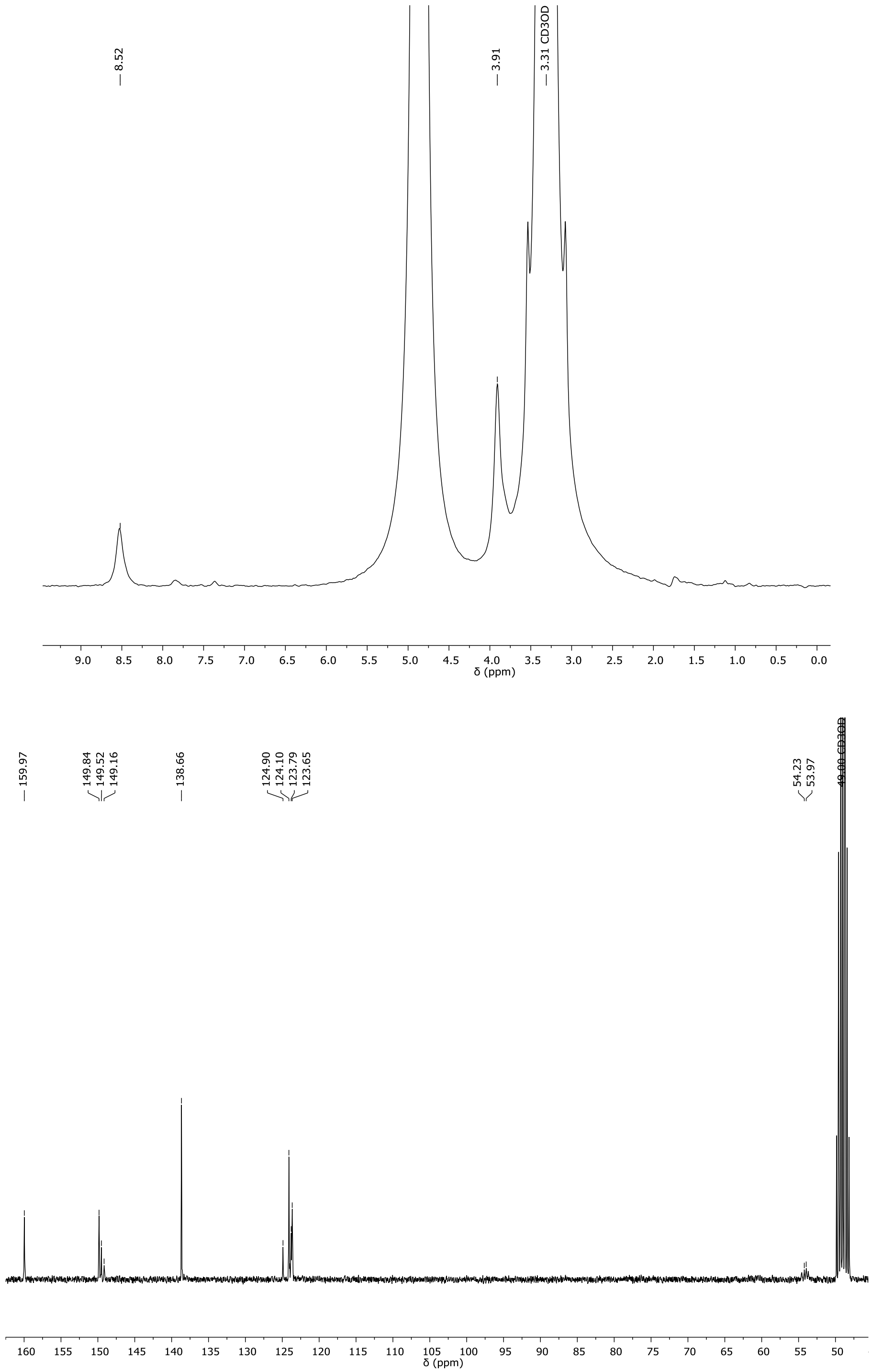

Figure S11. ^{1}H and ^{13}C NMR (300 MHz) of **TPA-*d*$_9$ + S-3** in methanol-*d*$_4$ at 298 K.
